# Divergent roles for Clusterin in Lung Injury and Repair

**DOI:** 10.1038/s41598-017-15670-5

**Published:** 2017-11-13

**Authors:** David M. Habiel, Ana Camelo, Milena Espindola, Timothy Burwell, Richard Hanna, Elena Miranda, Alan Carruthers, Matthew Bell, Ana Lucia Coelho, Hao Liu, Fernanda Pilataxi, Lori Clarke, Ethan Grant, Arthur Lewis, Bethany Moore, Darryl A. Knight, Cory M. Hogaboam, Lynne A. Murray

**Affiliations:** 1 Department of Medicine, Cedars-Sinai Medical Center, Los Angeles, CA USA; 2 Respiratory, Inflammation and Autoimmunity, MedImmune Ltd, Cambridge, United Kingdom; 3 Respiratory, Inflammation and Autoimmunity, MedImmune LLC, Gaithersburg, MD USA; 4 Translational Sciences, MedImmune LLC, Gaithersburg, MD USA; 5 Molecular Biology, MedImmune LLC, Gaithersburg, MD USA; 6 Translational Medicine, MedImmune LLC, Gaithersburg, MD USA; 7 Department of Internal Medicine, Division of Pulmonary and Critical Care Medicine, University of Michigan Health System, Ann Arbor, Michigan USA; 8 School of Biomedical Sciences and Pharmacy, University of Newcastle, Callaghan, NSW Australia

## Abstract

Lung fibrosis is an unabated wound healing response characterized by the loss and aberrant function of lung epithelial cells. Herein, we report that extracellular Clusterin promoted epithelial cell apoptosis whereas intracellular Clusterin maintained epithelium viability during lung repair. Unlike normal and COPD lungs, IPF lungs were characterized by significantly increased extracellular Clusterin whereas the inverse was evident for intracellular Clusterin. *In vitro* and *in vivo* studies demonstrated that extracellular Clusterin promoted epithelial cell apoptosis while intercellular Clusterin modulated the expression of the DNA repair proteins, MSH2, MSH6, OGG1 and BRCA1. The fibrotic response in Clusterin deficient (*CLU−/−*) mice persisted after bleomycin and it was associated with increased DNA damage, reduced DNA repair responses, and elevated cellular senescence. Remarkably, this pattern mirrored that observed in IPF lung tissues. Together, our results show that cellular localization of Clusterin leads to divergent effects on epithelial cell regeneration and lung repair during fibrosis.

## Introduction

Idiopathic pulmonary fibrosis (IPF) is a progressive lung disease characterized by a decline in lung function, destruction of lung architecture, deteriorating gas exchange, and increasingly non-elastic lungs. Median survival for patients is around three years following diagnosis^1^. Although the etiology of IPF remains elusive, genotoxic environmental irritants such as cigarette smoke and pollutants contribute to repeated and chronic damage to lung epithelial progenitors and cells^2,3^. This damage induces a cascade of pro-inflammatory and pro-fibrotic cytokines, such as transforming growth factor β (TGFβ), which promotes the activation and proliferation of fibroblasts and myofibroblasts, resulting in extensive deposition of extracellular matrix proteins, and an unending wound healing response^4^. Among many features present in IPF, it is hypothesized that epithelial progenitor exhaustion, type I alveolar epithelial cell (AEC) destruction, type II AEC hyperplasia^5^ predisposes the lung to aberrant remodeling. However, mechanisms leading to epithelial cell injury, exhaustion, and senescence remain elusive.

Clusterin (also known as apolipoprotein J) is a highly conserved glycoprotein whose gene expression is ubiquitous and up-regulated following exposure to cytotoxic stimuli^6^. The Clusterin gene yields two alternatively-spliced variants that encode for either a nuclear or a secreted protein^7^ with different biological functions. The nuclear form encodes for a 50 kDa protein that modulates DNA repair whereas the secreted form, a 75–80 kDa disulfide-linked heterodimeric protein, has been shown to have several functions including the clearance of apoptotic debris^7–11^. In the lung, Clusterin expression is up-regulated, where it promotes vascular remodelling in an animal model of pulmonary hypertension^12^. Clusterin has also been linked to oxidative stress in both asthma, COPD^13,14^ and renal fibrosis^15^. These studies prompted us to investigate its role in pulmonary fibrosis.

Herein, we characterized the role of Clusterin in clinical and experimental pulmonary fibrosis. Transcriptomic analysis indicated that there was increased *CLU* expression in IPF compared with COPD and normal lungs. Clusterin protein was significantly elevated in circulation but was significantly diminished inside epithelial cells in IPF lungs compared with COPD and normal healthy individuals. Exogenous Clusterin was pro-apoptotic in Clusterin deficient human epithelial cells especially in the presence of a genotoxic stressor. Further, knockdown of Clusterin via shRNA demonstrated an important, non-redundant, role for Clusterin in DNA repair within these cells. Indeed, transcriptomic analysis, immunohistochemical (IHC), and flow cytometric analysis of IPF lung showed a loss of expression of Clusterin and components of the Mismatch Repair (MMR), oxidative DNA damage repair and double strand break (DSB) repair pathways in epithelial cells in both the airway and honeycombed regions in IPF lungs. Finally, Clusterin deficient (*CLU−/−*) mice were protected from intrapulmonary bleomycin at Day 14 but had worse chronic lung fibrosis at Day 28. At the later time point, increased oxidative DNA damage, reduced expression of oxidative DNA repair components, MSH2, MSH6 and OGG1, and increased expression of senescent cell associated markers were evident in the *CLU−/−* compared with the wildtype group. Taken together our data demonstrate that Clusterin regulates DNA repair in response to DNA damaging agents, in which the loss of Clusterin led to chronic DNA damage and the senescence-associated responses in the epithelium potentially predisposing these cells and their progenitors to exhaustion and disrepair.

## Results

### Altered expression of Clusterin in lung fibrosis

**Figure 1:**
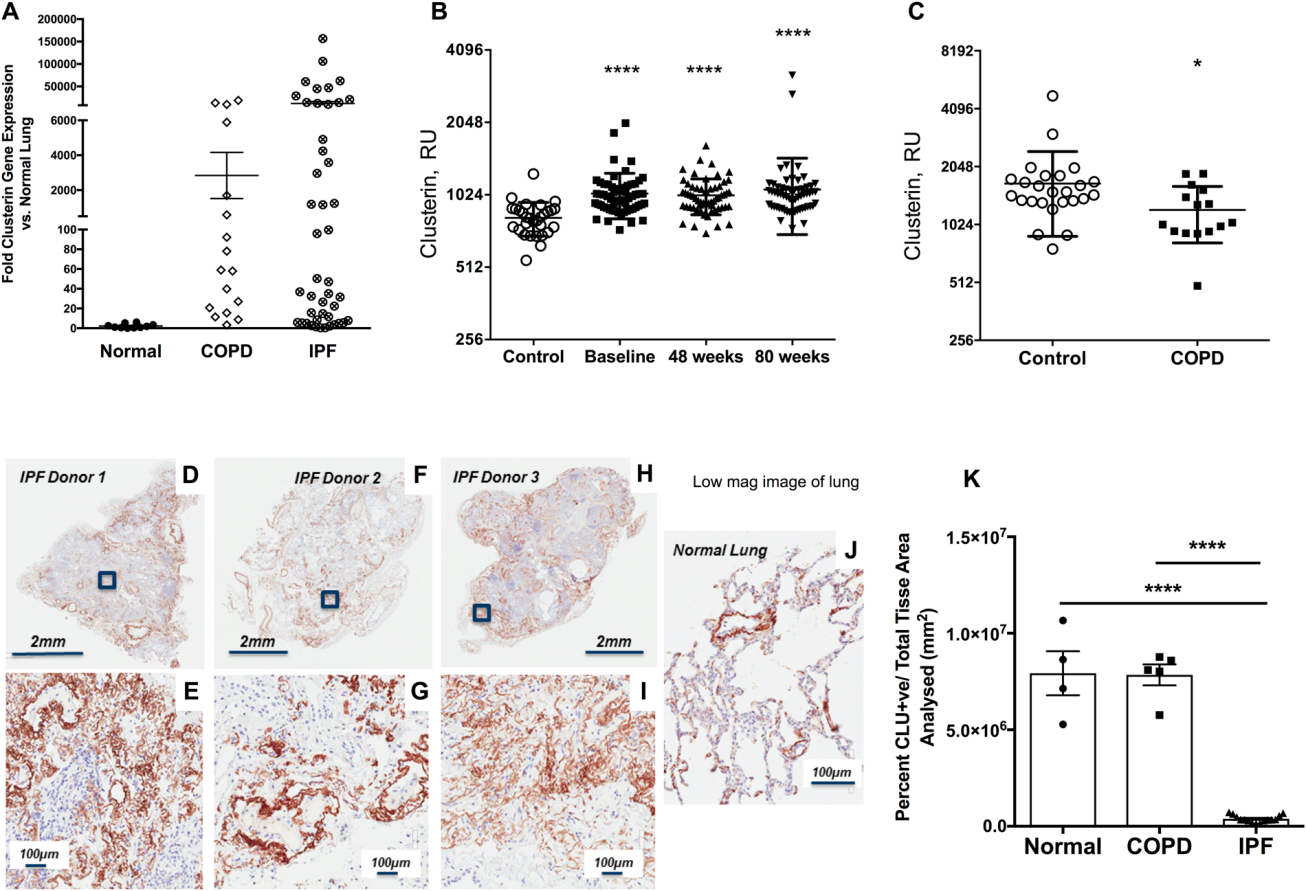
Elevated extracellular and reduced cell associated Clusterin in Idiopathic Pulmonary Fibrosis. (**A**) Clusterin gene expression was quantitated using RT-PCR in lung tissue from healthy control lung tissue (n = 10), COPD patients (n = 19) and IPF patients (n = 54). (**B,C**) Circulating Clusterin protein levels were quantitated and compared between IPF (n = 60) and a cohort of age matched controls (n = 30) (**B**), and from COPD (n = 15) and a separate cohort of age matched controls (n = 25) (**C**). Levels were measured by Somascan analysis, each dot representing a different individual. (**D–J**) Clusterin expression was visualized (brown staining) by IHC analysis of three IPF lungs (**D**–**I**) and a representative normal lung (**J**) tissue, size bars are indicated on image. (**K**) The staining intensity of cell-associated Clusterin was quantified in airway epithelial cells using Aperio Scanscope software. Shown is the average Clusterin staining intensity in airway epithelial cells in normal, IPF and COPD lung tissue. Data are expressed as Mean ± SEM *P ≤ 0.05, ****P ≤ 0.001 significance to relevant control levels.

IPF is associated with epithelial cell stress and injury. Consistent with previous observations of Clusterin upregulation in response to cellular stress^13,14,16–18^, transcriptomic analysis indicated increased *CLU* expression in the lungs of a subset of IPF patients compared with COPD and healthy control lungs (Fig. 1A). Longitudinal analysis of Clusterin levels in the circulation of IPF patients indicated that this protein was significantly elevated at various times after diagnosis compared with blood samples from healthy age-matched controls (Fig. 1B). There was significantly reduced levels of secreted circulating Clusterin in COPD compared with healthy age-matched controls (Fig. 1C), suggesting that increased Clusterin in the circulation was specific to IPF. Mining of publicly available RNA-sequencing datasets for Clusterin expression in normal human (Figure S1A) and mouse (Figure S1B) lung associated immune and structural cells suggested that this protein is expressed by the epithelial, endothelial and mesenchymal cells. IHC analysis showed that lung-associated Clusterin in IPF was detected predominantly within areas rich in elastin fibers (Figs 1D–J and S2A–H). In normal lungs, Clusterin predominantly immunolocalized to airway epithelial cells and was present in elastin-rich areas (Fig. 1J). IHC analysis followed by quantification of intracellular Clusterin staining indicated a loss of intracellular Clusterin protein in IPF compared with Normal and COPD airway epithelial cells (Fig. 1K). Indeed, mining of single cell RNA sequencing datasets^19^ showed a loss of Clusterin transcript in a subpopulation of indeterminate (Figure S3A) and basal (Figure S3B) but not Club/goblet cells from IPF lung explants (Figure S3C). However, there was no correlation between baseline Clusterin protein levels and Age (Figure S4A), baseline DLCO (Figure S4B), baseline FVC (Figure S4C), 80-week DLCO (Figure S4D) or 80-week FVC (Figure S4E) in IPF patients. Finally, Ingenuity Integrated Pathway Analysis (IPA) of transcriptomic datasets from laser-microdissected epithelial cells adjacent to fibroblastic foci, compared with normal areas of the same lung sample showed a reduction of Clusterin and many of its cell-associated interacting mediators (Figure S5). Together, these results suggested that secreted Clusterin was increased and epithelial cell-associated Clusterin was decreased in IPF.

### Extracellular Clusterin supplementation does not modulate bleomycin-induced lung fibrosis

**Figure 2:**
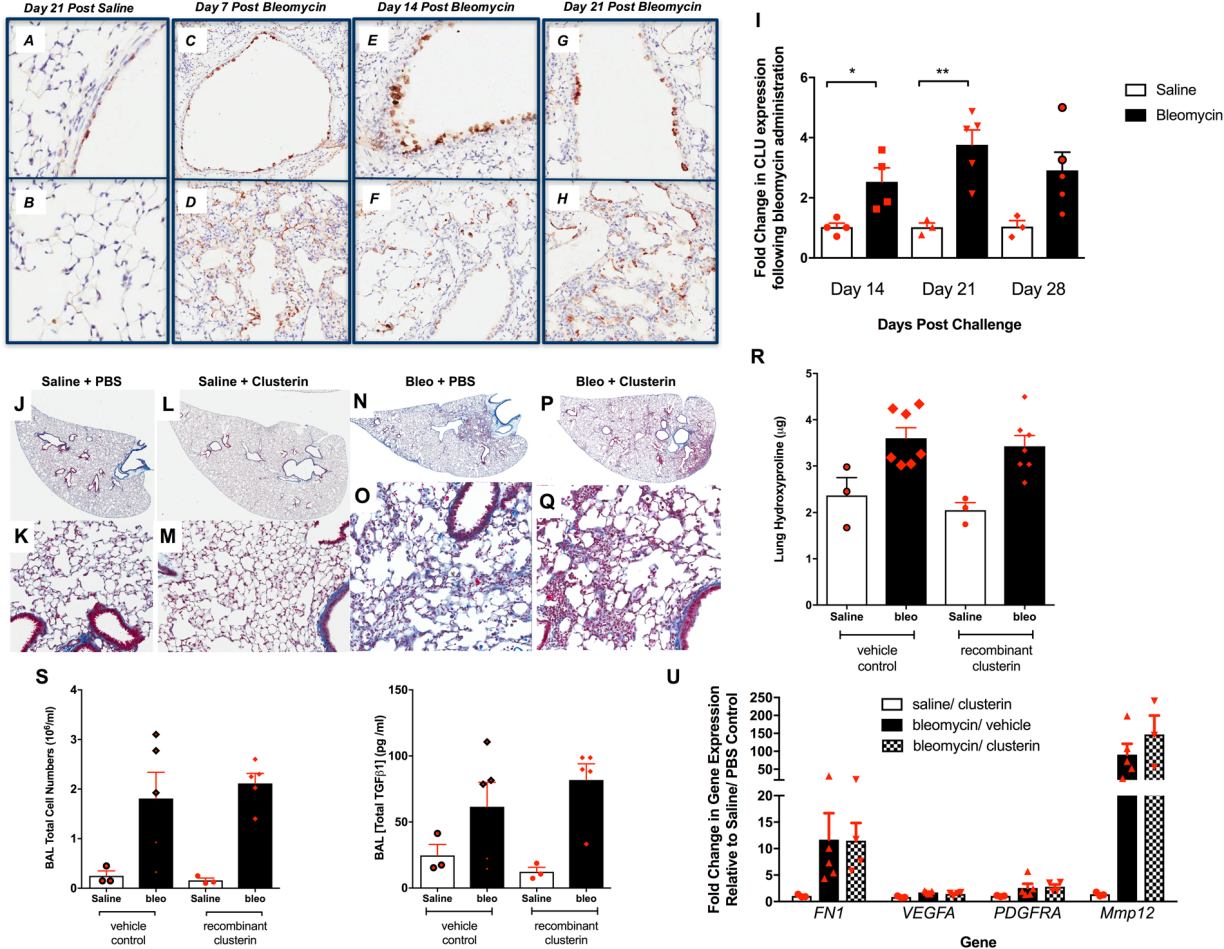
Induction of Clusterin in response to bleomycin-induced lung injury. (**A–H**) C57Bl/6 mice were given bleomycin intratracheally and *CLU* expression was visualized by IHC after 7 (**C**,**D**), 14 (**E**,**F**) and 21 (**G**,**H**) days of bleomycin instillation and compared to staining in the lungs of Day 21 saline treated mice (**A**,**B**). (**I**) Clusterin transcript expression was quantitated after 14, 21 and 28 days of bleomycin instillation by RT-PCR in whole lung samples. Data are expressed as Mean ± SEM*P ≤ 0.05, **P ≤ 0.01 significance to relevant control levels. (**J–Q**) Saline or bleomycin challenged C57Bl/6 mice were treated with 20 µg of recombinant Clusterin every three days starting at Day 2. Shown are representative histological images of the lungs stained with Masson’s trichrome from saline + PBS vehicle (**J**,**K**), saline + Clusterin (**L**,**M**), bleomycin + PBS (**N**,**O**) and bleomycin + Clusterin (**P**,**Q**) treated mice. The top images are of whole scans of the lungs and the bottom images are taken at 200x magnification. (**R**) Collagen content in the lungs of the recombinant Clusterin and vehicle treated mice was biochemically quantified using a hydroxyproline assay. Shown is the average hydroxyproline (µg) from right lung lobes. (**S**,**T**) Shown is the average total BAL cell number (**S**) and the total TGFß levels (**T**) in the lungs of vehicle or Clusterin treated mice 15 days after bleomycin challenge. (**U**) *FN1, VEGFA, PDGFRA* and *MMP12* transcript expression was quantified in whole lung samples 15 days after bleomycin, vehicle and/or Clusterin treatment. Shown is the average expression from 3 Saline/PBS, 3 Saline/rClusterin, 7 Bleo/PBS and 7 Bleo/rClusterin treated mice.

IHC analysis of saline treated murine lungs confirmed the localization of Clusterin protein in bronchial epithelial and interstitial cells (Fig. 2A,B). In bleomycin-challenged lungs, Clusterin immunopositive cells were more numerous and staining was localized in airway epithelial and interstitial cells in fibrotic regions at Days 7 (Fig. 2C,D), 14 (Fig. 2E,F), and 21(Fig. 2G,H) after bleomycin. Further, peak intracellular expression was observed at Day 14 after bleomycin (Fig. 2E,F), when the BAL levels of this protein averaged between 8–10 µg/ml (Figure S6). Consistent with the IHC analysis, transcriptomic analysis indicated that Clusterin expression was significantly elevated in bleomycin-challenged murine lungs at Days 14 and 21, compared with saline-treated mice (Fig. 2I).

Given the presence of 8–10 µg/ml of Clusterin in the BAL from 14-day bleomycin challenged wildtype mice (Figure S6), experiments were designed to determine the potential role of secreted Clusterin in modulating lung injury and repair in response to bleomycin. Saline- and bleomycin-challenged mice were intranasally treated with 20μg/dose of recombinant Clusterin (rClusterin, approximately twice the amount detected in the BAL after 14 days of bleomycin) every 3 days starting at Day 2 post-bleomycin and compared to animals treated with PBS. Recombinant Clusterin treatment of saline or bleomycin challenged mice did not alter collagen levels in the lungs compared to the vehicle (PBS) treated counterparts, as assessed by Masson’s Trichrome histological staining (Fig. 2L,M,P,Q vs J,K,N,O, respectively) and biochemical hydroxyproline quantification (Fig. 2R). Further, rClusterin did not modulate total BAL cell counts (Fig. 2S), TGFβ1 protein levels (Fig. 2T) or *FN1, VEGFA, PDGFRA*, and *MMP12* transcript levels compared with the control group (Fig. 2U). Finally, a transcriptomic analysis of factors known to play a role in lung injury and remodeling indicated that rClusterin didn’t modulate any of these genes (Figure S7A–F). Together, these results suggest that the addition of exogenous Clusterin protein did not modulate the fibrotic responses to bleomycin in murine lungs.

### Persistence of bleomycin-induced fibrosis in Clusterin deficient mice

**Figure 3:**
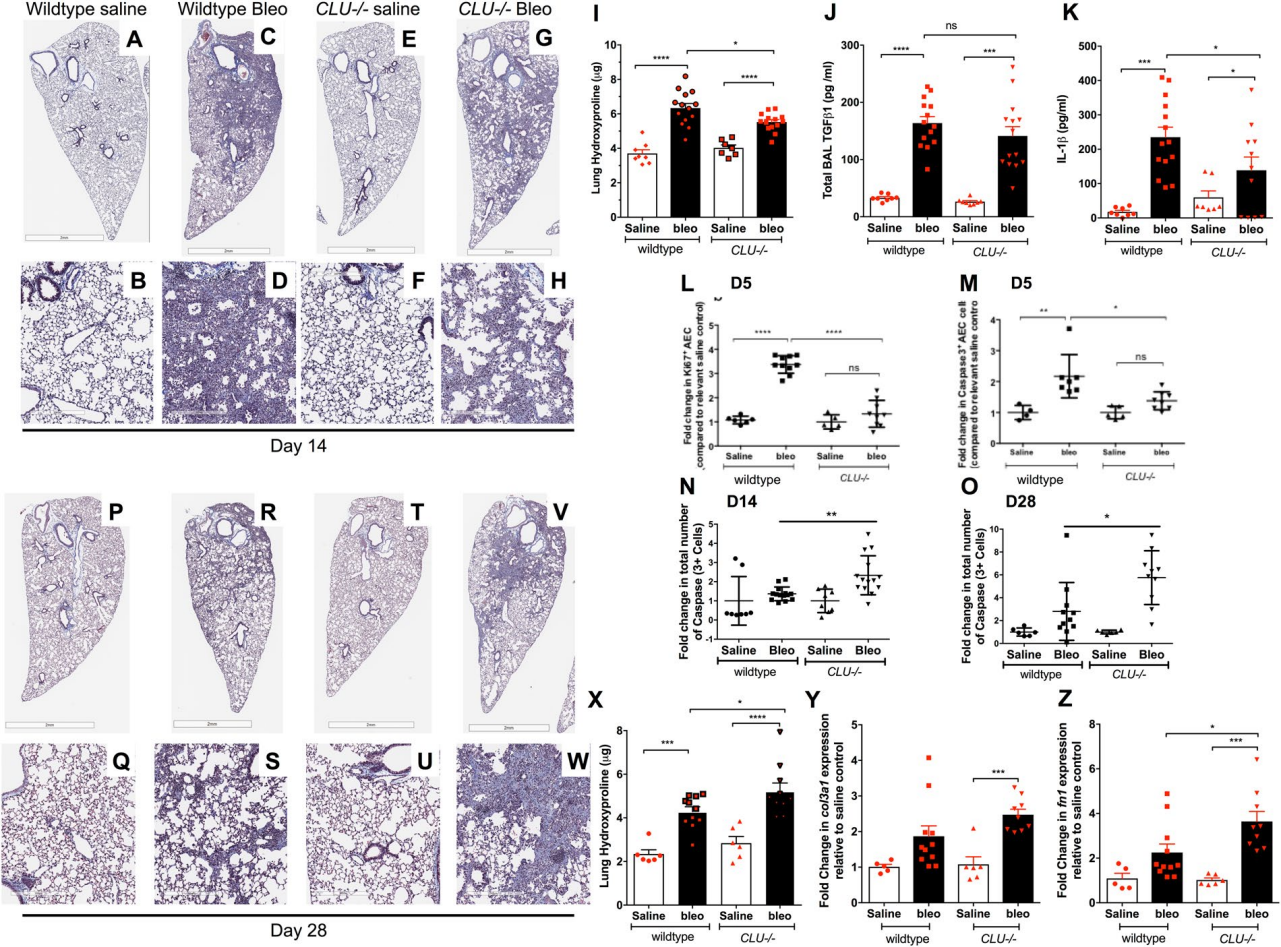
Clusterin deficient mice show persistent fibrosis in response to bleomycin. WT or *CLU −/−* received bleomycin intratracheally on Day 0. (**A–H**) Depicted is Masson’s Trichrome histological staining of WT saline (**A**,**B**), WT bleomycin (**C**,**D**), *CLU−/−* saline (**E,F**) and *CLU−/−* bleomycin (**G**,**H**) treated lungs fourteen days after instillation. Shown are whole lung images (top) and magnified images (bottom). (**I**) Collagen content in the lungs of the of bleomycin treated wildtype and *CLU−/−* mice was biochemically quantified using a hydroxyproline assay, 14 days after bleomycin instillation. Shown is the average hydroxyproline (µg) from right lung lobes. (**J**) Shown is the average total TGFβ levels in the BAL from wildtype and *CLU−/−* mice, 14 days after bleomycin challenge. (**K**) Shown is the average IL-1β levels in whole lung lysates from wildtype and *CLU−/−* mice, 14 days after bleomycin challenge. (**L**,**M**) The proliferative status (**L**) and Caspase3 expression (**M**) in live CD45^−^ EpCAM^+^ epithelial cells was assessed via Ki67 and activated Caspase-3 staining, respectively, 5 days after bleomycin challenge. Depicted is the fold change in cell number in response to bleomycin, relative to its relevant saline control. (**N**,**O**) IHC analysis for cleaved Caspase3 was performed on fibrotic murine lungs and quantified using Aperio Scanscope software. Depicted is the fold change in clusters, as defined by three or more adjacent positive cells, staining for cleaved Caspase3, 14 (**N**) and 28 (**O**) days after bleomycin challenge, compared to relevant saline control. (**P**–**W**) Depicted is Masson’s Trichrome histological staining of WT saline (**P**,**Q**), WT bleomycin (**R**,**S**), *CLU−/−* saline (**T,U**) and *CLU−/−* bleomycin (**V**,**W**) treated lungs 28 days after instillation. Shown are whole lung images (top) and magnified images (bottom). (**X**) Collagen content in the lungs of the of wildtype and *CLU−/−* treated mice was biochemically quantified using a hydroxyproline assay 28 days after bleomycin instillation. Shown is the average hydroxyproline (µg) from right lung lobes. (**Y**,**Z**) Quantitative PCR analysis was performed on RNA purified from lungs of 28-day saline and bleomycin treated murine lungs. Shown is the average *col3a1* (**Y**) and *fn1* (**Z**) transcript expression. Data are Mean ± SEM., n = 8–13 mice/ group. *P ≤ 0.05, **P ≤ 0.01, ***P ≤ 0.005, ****P ≤ 0.001 significance, or as stated. ns = not significant.

At Day 14 after bleomycin, Clusterin deficient (*CLU−/−*) mice had significantly reduced lung hydroxyproline levels (Fig. 3I) and histological collagen staining as assessed via Masson’s Trichrome staining (Fig. 3A–H). While there was no difference in total TGFβ1 protein levels in BAL between the wildtype and *CLU−/−* groups (Fig. 3J), there was a reduction in whole lung IL-1β protein levels, another well-known pro-fibrotic cytokine, in *CLU−/−* groups (Fig. 3K). Flow cytometric analysis of single cell suspensions from lung tissue at Day 5 after bleomycin indicated that proliferating Ki67^+^ epithelial cells (AECs; as defined by CD45 negative, EpCAM^+^ cells) (Fig. 3L) and Caspase 3^+^ AECs (Fig. 3N) were significantly decreased in *CLU−/−* mice versus wild-type mice. However, there was a significant increase in the number of cleaved-Caspase3^+^ cells between wildtype and *CLU−/−* mice, 14-days (Fig. 3N) and 28-days (Fig. 3O) after bleomycin administration.

Depending on the dose of bleomycin used, the fibrosis induced by bleomycin challenge begins to diminish by Day 28 after challenge^20^. Therefore, we next analyzed the extent of the fibrotic response in both *CLU−/−* and wildtype groups at Day 28 post-bleomycin. Consistent with the elevated number of apoptotic cells (Fig. 3O) at this time point, lung hydroxyproline levels (Fig. 3X) and histological collagen staining via Masson’s Trichrome staining (Fig. 3P–W) was significantly elevated in the *CLU−/−* relative to wildtype mice. Both *col3a1* (Fig. 3Y) and *fn1* (Fig. 3Z) transcripts were significantly elevated in the *CLU−/−* relative to wildtype mice. We also assessed the expression of two genes typically associated with alveolar repair, namely *vegfa* and *pdgfra* ^21^ and observed that bleomycin-challenged *CLU−/−* mice had significantly reduced *vegfa* (Figure S8A) and *pdgfra* (Figure S8B) transcripts at Day 28 compared with saline controls. Thus, these results suggest that Clusterin is required for the resolution of lung fibrosis.

### Exogenous Clusterin induces the expression of cell cycle arrest-associated CDKN1B and CDKN2A and promotes the apoptosis of human bronchial epithelial cells

There are several known variants of Clusterin protein that are expressed during cellular stress via alternative promoter start sites and/or alternative splicing^22^. In particular, various isoforms of the protein may modulate DNA repair pathways under genotoxic stress^8,9,11^. To determine the fate of extracellular Clusterin, rClu protein was fluorescently labeled and exogenously added to HBECs. Exogenously added rClu protein was readily taken up by HBECs over 4 hours, where this protein predominately localized in the cytoplasm (Figure S9A–C). Further, in shRNA Clusterin-knocked down HBECs showed a slight elevation of Caspase 3/7 activity and the addition of 10 µg of rClu further increased caspase 3/7 activation compared with scrambled RNA treated cells (Figure S9D). Clu deficient cells showed a significant reduction of Caspase 3/7 activation after H2O2 challenge, relative to cluster proficient cells (Figure S9D), which was completely abrogated by the addition of exogenous Clusterin to the Clu deficient cells. Finally, H2O2 chalenged *Clu* deficient cells had elevated cell-cycle arrest associated *CDKN1B* and *CDKN2A* transcripts, relative to *Clu* proficient cells and addition of exogenous rClu did not modulate these transcripts (Figure S9E,F). However, elevated IL1B transcript in H2O2 treated clusterin deficient cells was abrogated by the addition of exogenous rClu (Figure S9G). Together, these results demonstrate that extracellular Clusterin may enhance the apoptotic cell death; and that Clusterin protein expression might be required to reduce epithelial cell senescence in the presence of genotoxic stimuli.

### Cell-associated Clusterin regulates DNA repair pathways in human epithelial cells

To further assess the mechanism through which intracellular Clusterin modulates HBEC response to genotoxic stress, Clusterin expression was targeted using shRNA in primary bronchial epithelial cells *in vitro* followed by RNA sequencing analysis. Knockdown efficiency was confirmed, where there was a greater than 70% reduction in *CLU* mRNA expression and 50% reduction in Clusterin protein levels (data not shown). Ingenuity IPA analysis indicated that shRNA-mediated knockdown of Clusterin resulted in a major loss in the expression of components from various DNA repair pathways (Table S1), including Mismatch Repair in Eukaryotes (Figure S10) and Role of BRCA1 in DNA Damage Response (Figure S11) ingenuity canonical pathways. Consistent with the finding that chronic DNA damage is known to induce cellular senescence^23^, ingenuity upstream regulator analysis indicated that the loss of Clusterin led to the activation of various upstream regulators commonly associated with stress responses and senescence (Table S2), including NUPR1, CDKN2A, IL1β, Rb, and TP53. Thus, these results demonstrated that cell-associated Clusterin might be required for optimal DNA repair, particularly in lung epithelial cells.

### Bleomycin-induced lung injury in CLU−/− mice leads to increased DNA damage and cellular senescence

**Figure 4:**
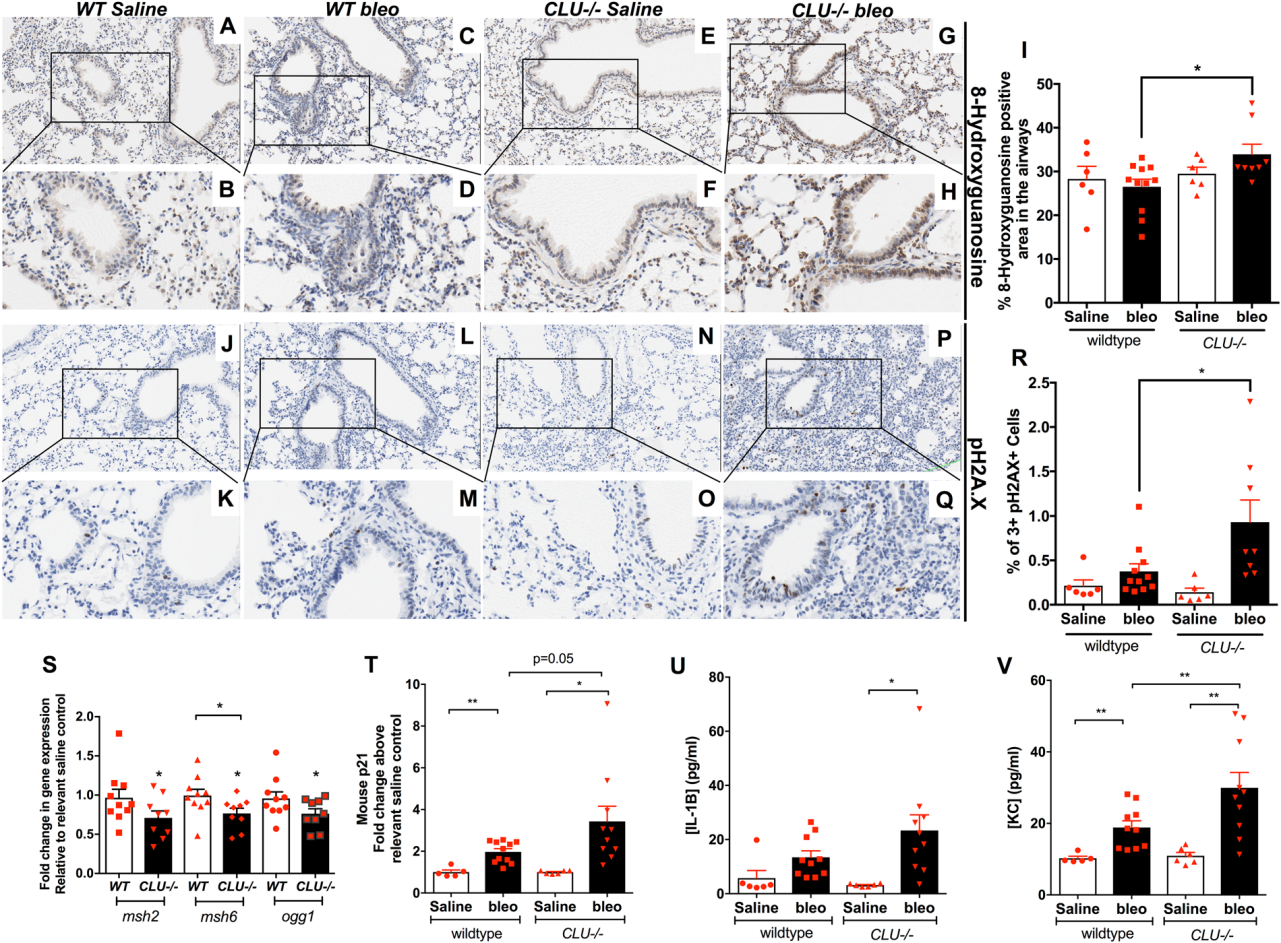
CLU*−/−* show increased oxidative DNA damage, decreased MSH2, MSH6 and OGG1 transcript expression and increased expression of senescence markers. Twenty-eight days after bleomycin instillation in wildtype and *CLU−/−* mice, lungs were histologically stained for various markers for DNA damage. (**A–H**) Depicted are representative images for wildtype saline (**A**,**B**), wildtype bleomycin (**C**,**D**), *CLU−/−* saline (**E**,**F**) and *CLU−/−* bleomycin (**G**,**H**) treated lungs stained for the oxidative DNA adduct, 8-Oxo-2′-deoxyguanosine (8-Oxo-dG). (**I**) Depicted is 8-Oxo-dG staining intensity in airway epithelial cells as determined using Aperio Scanscope software in saline and bleomycin treated wildtype and *CLU−/−* mice. (**J–Q**) Depicted are representative images for wildtype saline (**J,K**), wildtype bleomycin (**L**,**M**), *CLU−/−* saline (**N**,**O**) and *CLU−/−* bleomycin (**P**,**Q**) treated lungs stained for DNA double-strand break associated histone, phospho-H2A.X. (**R**) Depicted is the percent of cell clusters, as defined by three or more adjacent positive cells, staining for p-H2A.X, as determined using Aperio Scanscope software in saline and bleomycin-challenged wildtype and *CLU−/−* mice. (**S**) Depicted is the expression of *Msh2, Msh6* and *Ogg1* transcripts in whole lungs from wildtype and *CLU−/−* knockout mice, 28 days after bleomycin instillation and compared to relevant saline controls. (**T**–**V**) ELISAs were performed to measure the concentration of p21, IL-1β and KC on whole murine lung tissues. Depicted is the fold change of p21 (**T**) and the pg/ml of IL-1β (**U**) and KC (**V**) protein concentration in the lungs from wildtype and *CLU−/−* mice, 28 days after saline or bleomycin challenge. n = 8–13 mice/ group. *P ≤ 0.05, **P ≤ 0.01 significance, or as stated. ns = not significant.

To determine whether Clusterin regulates DNA repair pathways and DNA damage associated senescence *in vivo* following bleomycin challenge, qPCR and IHC analysis were performed for the oxidized DNA adduct, 8-Oxo-2′-deoxyguanosine (8-Oxo-dG), DNA double strand break associated phospho-histone H2AX (p-H2A.X), the senescent-associated proteins, p21 and IL-1β, and various components of oxidative DNA damage repair pathways (*msh2, msh6 & ogg1)* in wild type and *CLU−/−* mice at Days 14 and 28 after bleomycin challenge. After 14-days of bleomycin administration, both bleomycin-treated wildtype and *CLU−/−* mice had increased percent p-H2A.X^+^ cell clusters, as defined by clusters of 3 or more positive cells, but there were no significant differences between the genotypes (Figure S12A). Further, there was no difference in either 8-Oxo-dG intensity (Fig. 4A–D; quantified in 4I) or percent p-H2A.X^+^ cell clusters, (Fig. 4J–M; quantified in 4R), 28-days after saline and bleomycin-treatment of wildtype mice. However, there was a significant increase in 8-Oxo-dG staining intensity (Fig. 4C,D vs. G,H and quantified in Fig. 4I) and p-H2A.X^+^ cell clusters (Fig. 4L,M vs. P,Q and quantified in Fig. 4R), in *CLU−/−* compared to wildtype mice, 28-days after bleomycin-challenge. Finally, exogenously administered Clusterin protein to saline or bleomycin treated wildtype mice did not modulate the number of p-H2A.X^+^ cell clusters (Figure S12B) or the percentage of 8-Oxo-dG positive cells in the airways (Figure S12C).

To identify mechanisms leading to these changes in wildtype and *CLU−/−* mice, we next examined the expression of DNA repair components. There was no difference between saline-treated and bleomycin-treated wildtype mice for the expression of *msh2*, *msh6*, or *ogg1*; however, there was a significant decrease in the expression of *msh2*, *msh6*, and *ogg1* transcripts in *CLU−/−* bleomycin-challenged mice compared to *CLU−/−* saline-control mice (Fig. 4S). Moreover, there was a significant decrease in *msh6* in *CLU−/−* compared to wildtype mice following bleomycin challenge at Day 28. There was an increase in whole lung p21 and IL1ß proteins (Fig. 4T,U) and KC transcript (Fig. 4V) expression in the bleomycin-challenged *CLU−/−* compared with their wildtype counterparts at this time point. Finally, there was a significant increase in *sftpc* transcript expression in wildtype but not *CLU−/−* mice, 28 days after bleomycin administration (Figure S13). Together, these results support our *in vitro* results and suggest that cell-associated Clusterin in required for oxidative DNA damage repair and epithelial regeneration.

### Clusterin and its associated DNA repair proteins are downregulated in IPF basal epithelial cells

**Figure 5:**
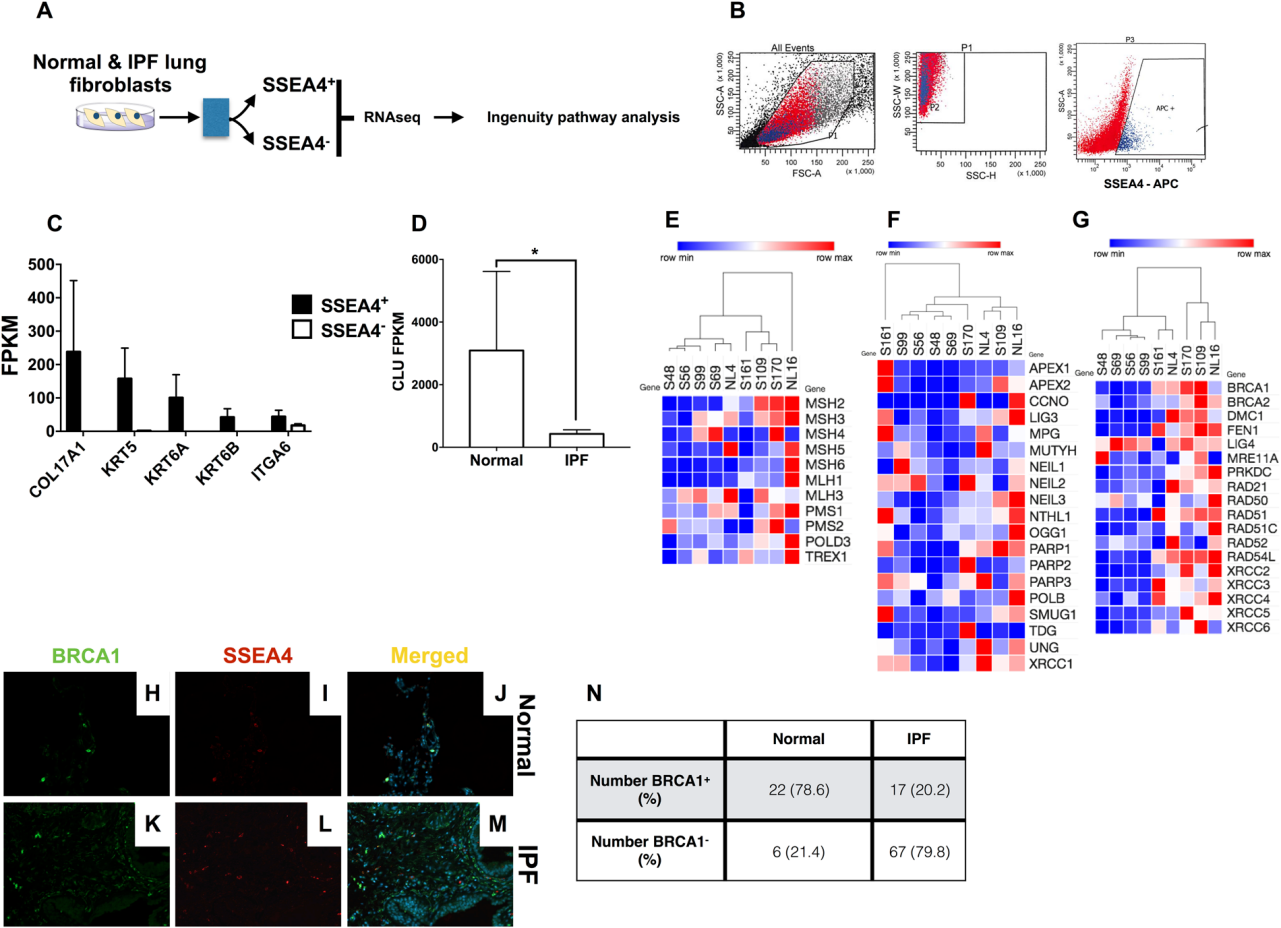
Loss of transcripts for Clusterin, MMR, BER & DSB DNA repair pathway components in IPF SSEA4^+^ basal-like epithelial cells. (**A**) SSEA4^+^ and SSEA4^−^ cells were sorted from normal and IPF stromal cultures, RNA was extracted and subjected to RNAseq analysis. Fold changes in IPF relative to normal were calculated and the results were uploaded onto ingenuity IPA for pathway analysis (**A**). (**B**) Shown is an representative sort plot depicting the forward and side scatter localization of SSEA4^+^ cells. (**C**) Normalized RNA sequencing FPKM values were mined for transcripts that were enriched in SSEA4^+^ relative to SSEA4^−^ cells. Shown are enriched basal cell lineage markers in normal lung and IPF SSEA4^+^ cells. (**D**) Shown is the average *CLU* transcript FPKM values normal and IPF SSEA4^+^ cells. (**E–G**) Transcripts encoding for components of MMR (**E**), BER (**F**) and DSB (**G**) pathways were mined from SSEA4^+^ RNAseq data, clustered using Euclidean distance with complete linkage and heat maps were generated using Morpheus (Broad Institute). (**H–M**) Normal and IPF lung biopsies were stained with anti-SSEA4 and BRCA1 antibodies followed by fluorescent microscopic analysis. Representative images were taken at 200x magnification from two normal and four IPF lung biopsies stained with BRCA1 (**H**,**K** respectively), SSEA4 (**I**,**L** respectively) and the merged composites (**J**,**M** respectively). (**N**) The number of SSEA4^+^ cells showing both SSEA4 and BRCA1 staining was quantified, averaged, and the percentage of cells was calculated from 28 normal and 84 IPF SSEA4^+^ cells from 3 normal and 4 IPF lungs, respectively.

To further examine the expression and role of intracellular Clusterin in other IPF cell types, recently described SSEA4^+^ progenitor cells^24^ and SSEA4^−^ stromal cells were flow sorted from normal and IPF lung-derived stromal cultures and subjected to RNA sequencing analysis, followed by Ingenuity IPA analysis (Fig. 5A). SSEA4^+^ cells were on average smaller and less granular than SSEA4^−^ cells as assessed by their forward and side scatter flow cytometric profile (Fig. 5B). Interestingly, SSEA4^+^ cells were enriched with transcripts commonly observed in airway epithelial basal cells^25^ (Fig. 5C). Further, there was a significant reduction in *CLU* expression (Fig. 5D) and a concurrent loss of transcripts encoding for components of the MMR (Fig. 5E), Base excision repair (BER; Fig. 5F), and DDR (Fig. 5G) pathways in IPF compared with normal SSEA4^+^ cells. Immunofluorescence analysis for BRCA1 and SSEA4 in IPF lung biopsies and normal lung explants supported the transcriptomic results, in which there was a loss of BRCA1 and SSEA4 colocalization in IPF but not normal lungs (Fig. 5H–M and quantified in Fig. 5N). Ingenuity upstream regulator analysis of IPF compared with normal SSEA4^+^ cells demonstrated that there was increased activity of senescent-associated upstream regulators including TP53, IL1, CDKN2A and RB1 (Table S3). These results support the *in vitro* Clusterin shRNA knockdown and murine studies and suggest that Clusterin might be required for the expression of various components of DNA repair pathways in lung epithelial cells.

### Increased 8-Oxo-dG nuclear staining and a loss of MSH2 and MSH6 in airway epithelial cells in IPF lungs

**Figure 6:**
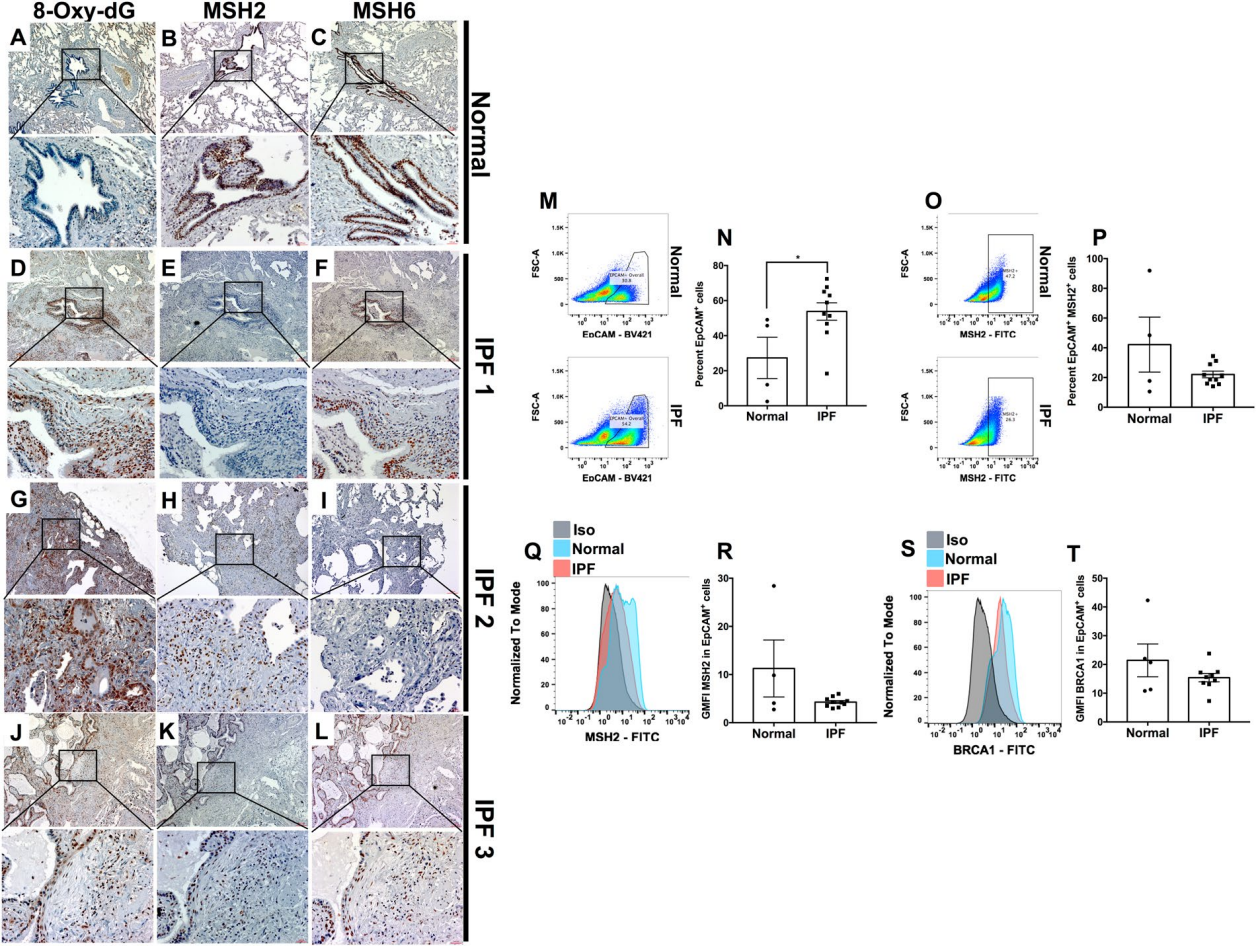
Increased nuclear 8-Oxo-dG and loss of the mismatch repair components, MSH2 and MSH6, in airway epithelial cells in IPF lungs. (**A–L**) Normal and IPF lung biopsies were stained with anti 8-Oxo-dG (**A** and **D**, **G** and **J**, respectively), anti MSH2 (**B** and **E**, **H** and **K**, respectively) and anti MSH6 (**C** and **F**, **I** and **L**, respectively). Shown above are representative images taken at 50x (top) and 200x (bottom) magnification from 3 normal and 10 IPF lungs. (**M–P**) Normal and IPF lung explants were mechanically dissociated and the resulting cellular suspensions were fixed, permeabilized and stained with anti-EpCAM and anti-MSH2 antibodies. Shown are representative flow cytometric dot plots for EpCAM^+^ (**M**) and MSH2^+^ cells within the EpCAM^+^ population (**O**) epithelial cells stained normal (top) and IPF (bottom) and the average percentage of cells showing EpCAM (**N**) and EpCAM and MSH2 (**P**) from 4 normal and 10–11 IPF lung explants. (**Q–T**) Shown are representative histograms depicting MSH2 and BRCA1 expression (**Q** and **S**, respectively) and GMFI (**R** and **T**, respectively) from 4 normal and 9 IPF lung explant derived EpCAM^+^ cells.

Given that our *in vitro* and *in vivo* studies suggested an important, non-redundant, role for Clusterin in the expression of various components of oxidative DNA damage repair pathways, we hypothesized that an abundance of oxidative DNA damage and loss of components of oxidative DNA repair pathways would be apparent in the Clusterin-deficient airway epithelial cells in IPF. To this end, 8-Oxo-dG was analyzed in IPF lung biopsies and explants and compared with normal lung explants. Increased 8-Oxo-dG histological staining was evident in epithelial cells, particularly in the airway, and honeycombing regions, and in epithelial cells adjacent to fibroblastic foci (Fig. 6D,G and J) whereas little 8-Oxo-dG was evident in normal lung explant tissue (Fig. 6A). Further, IHC analysis indicated that there was marked loss of the MMR components, MSH2, and MSH6, in all of the epithelial cell types (Fig. 6E,F,H,I and K,L) relative to normal airway epithelial cells (Fig. 6B,C). Flow cytometric analysis of lung explant cells showed an abundance of weakly adherent EpCAM^+^ cells in IPF compared with normal tissue (Fig. 6M,N). MSH2 and BRCA1 staining indicated that there was decreased MSH2 (Fig. 6O–R; percent positive and GMFI) and BRCA1 (Fig. 6S,T; GMFI) proteins in IPF compared with normal lung EpCAM^+^ cells. Taken together, our data demonstrates that Clusterin maintains oxidative DNA repair pathways thereby reducing the burden of DNA damage and DNA damage-induced senescence, which has a newly described role in pulmonary fibrosis^26^.

## Discussion

Clusterin (apolipoprotein J) is a pleiotropic protein that is induced during cellular stress^13,16,17^, and has extracellular roles, such as modulation of apoptosis^16,17,27,28^, and intracellular roles, where it modulates the removal of misfolded proteins and^8^ and DNA repair^8,9,11,29^. Variants of Clusterin are regulated via alternative promoter start sites and alternative splicing^22^, which ultimately leads to its secretion or retention in a cell. Given that IPF is characterized by pronounced epithelial cell apoptosis, loss of alveolar type I & II epithelial cells, bronchiolization of the alveoli and impaired repair^19^, we explored the expression and function of Clusterin in both experimental bleomycin-induced pulmonary fibrosis (in which Clusterin expression was altered in a time-dependent manner) and primary normal and IPF cells. In contrast to normal volunteers and COPD patients, both *CLU* expression in lung samples and secreted Clusterin protein in the circulation were elevated in IPF patients. Intracellular Clusterin was observed in the airway epithelium and around elastin fibers in normal lung tissue. IHC and transcriptomic analysis (both single cell^19^ and bulk analysis) suggested that this protein is lost in epithelial cells in IPF patients. Further, *in vitro* studies suggest that extracellular Clusterin can be internalized by epithelial cells, however, this was not observed in IPF lung tissue. This suggests that there may be a deficiency in the uptake of extracellular Clusterin by IPF epithelial cells. Alternatively this protein is not take up by cells *in vivo*.

The absence of intracellular Clusterin in IPF epithelial cells, was observed in hyperplastic epithelial cells that also showed diminished MSH2 and MSH6 DNA repair pathways. Consequently, we focused an IHC analysis on epithelial cells in the airway and honeycombing regions in IPF tissue samples. EpCAM was utilized as a pan-epithelial marker in our flow cytometric analysis to support our IHC analysis. While it is plausible that changes observed in IPF relative to normal EpCAM^+^ cells in our flow cytometric analysis might be due to differences in epithelial cell population present in these lungs, our IHC analysis on epithelial cells lining airways and honeycombing cysts were consistent with our flow cytometric results. The source of secreted Clusterin protein in IPF is not presently clear but analysis of publicly available single cell RNAseq datasets^19^ indicated that this protein is expressed by lung—associated epithelial, endothelial and mesenchymal cells in murine and human lungs. This suggest that other cell types in IPF lungs may likely contribute to the observed elevation in Clusterin proteins.

Although our studies are somewhat limited by the lack of selective tools for modulating extracellular vs. intracellular variants of Clusterin, we employed rClusterin (i.e. extracellular) and shRNA knockdown of this factor in cultured HBECs. The addition of rClusterin to HBECs increased Caspase 3/7 activation in untreated Clusterin deficient cells and did not rescue cell-cycle arrest associated *CDKN2A* and *CDKN1A* transcript expression in H2O2 challenged Clusterin deficient cells. *In vivo* deficiency of Clusterin protein induced a protective effect, 5 days after bleomycin administration but promoted apoptotic cell death, as evident by the abundance of cells expressing cleaved Caspase-3 protein, after 14 and 28 days of bleomycin. The protective effect observed early in this model may be due to the lack of Clusterin-induced apoptosis (as observed in our *in vitro* studies with exogenously added rClu); however the pro-apoptotic effect observed at later time points may be due to reduced DNA repair and persistent DNA damage in *CLU−/−* cells relative to their wildtype counterparts. Exogenous supplementation of Clusterin to wildtype mice in this model did not modulate apoptosis, lung fibrosis or the burden of double strand DNA breaks or oxidative DNA damage. We speculate that the elevated Clusterin protein present in wildtype mice likely negated any pro-apoptotic or genoprotective effect(s) of exogenous Clusterin in these mice following bleomycin-induced lung injury.

**Figure 7:**
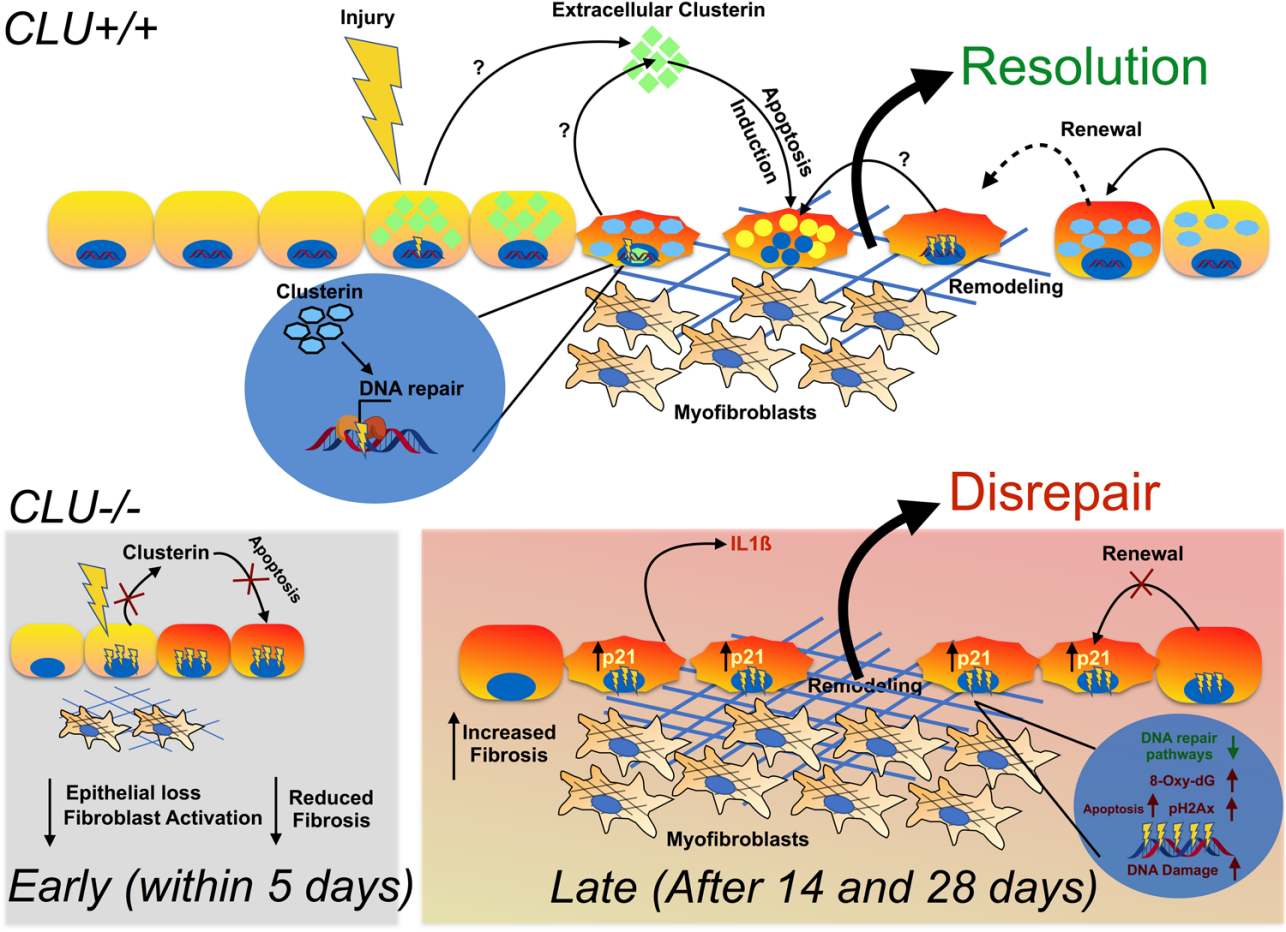
Pictorial summary for the potential role of Clusterin variants in the modulation of epithelial cell survival and lung fibrosis.

Targeting Clusterin using shRNA approaches in HBECs also revealed a potential role for intracellular Clusterin in modulating DNA repair pathway expression and function. These *in vitro* data were consistent with our findings at Day 28 after bleomycin when there was a significant increase in DNA double strand break foci and oxidative DNA damage, apoptotic cells and increased expression of cellular senescence markers p21, IL1ß and associated proinflammatory, KC, proteins. Collectively, we hypothesize that Clusterin might plays a dual role in in the lung depending on its primary localization: its extracellular and cytosolic localization might promote epithelial cell apoptosis while its nuclear localization might be potentially reqired for efficient DNA repair and regeneration particularly after exposure of these cells to genotoxic mediators (Summarized in Fig. 7). Given that Clusterin is primarily localized extracellularly in IPF, one might speculate that this could be a mechanism driving increased epithelial cell apoptosis in this disease but future work is warranted to better characterize the effects of Clusterin variants in epithelial cell apoptosis, DNA repair, and senescence.

Senescence is a phenomenon that has been associated with various physiological processes including telomere uncapping, chronic DNA damage and oncogene activation. In healthy tissue, senescence is linked to the appropriate clearance of apoptotic cells^30^. However, in disease, the presence of senescent cells has been linked to premature aging, where there is an accumulation of damaged cells, which can trigger inflammation and aberrant remodeling. Senescence has been reported as an active component in IPF^31^ and in the bleomycin model of pulmonary fibrosis^32,33^. A role for Clusterin in tumor cell senescence has been reported (reviewed in^34^), however its role in senescence in other cell types is less clear. Our *in vitro* studies showed that Clusterin deficiency promoted the expression of cell cycle arrest- and senescence-associated *CDKN1B, CDKN2A* and *IL1B* transcripts in H2O2 challenged cells. This was partially rescued by the addition of exogenous rClu protein, where Clu deficient HBEC-associated *IL1B* transcripts were markedly reduced in the presence of exogenous rClu. Further, our transcriptomic data of Clusterin deficient HBECs indicated that the lack of Clusterin led to the expression and activation of various proteins and upstream regulators commonly observed in senescent cells in primary HBECs. Finally, elevated p21 protein expression and other senescence associated markers at Day 28 after bleomycin challenge was observed in *CLU−/−* mice compared with their wildtype counterparts. One potential mechanism leading to cellular senescence in cells lacking Clusterin is the reduction in the expression and activity of components of various DNA repair pathways, including MSH2, MSH6, BRCA1 and/or OGG1. Indeed, it is likely that this senescence phenotype is contributing to impaired tissue repair as recently shown in aged mice with a high senescent burden^26^.

Mechanisms linking Clusterin to the expression of MSH2, MSH6, BRCA1 and/or OGG1 and its role in oxidative DNA repair remain elusive; however, various reports have shown that nuclear Clusterin interacts with the double strand break repair component Ku70^8,11^. Further, the mismatch repair component, MSH6, has been observed to interact with both MSH2^35^ and Ku70^36^, where its latter interaction contributed to double-strand break repair. Indeed, reduced Ku70 function have been linked to aging and senescence^37^, supporting a potential role for cell-associated Clusterin in the induction of cellular senescence. This suggests that in addition to modulating the expression of various DNA repair components, cell-associated Clusterin might also directly modulate the activity of these pathways. Future experiments are warranted to better characterize the role of cell-associated and secreted Clusterin in oxidative DNA damage repair.

Collectively, our data demonstrate that Clusterin has divergent roles in both human epithelial cells and murine lungs, following their exposure to the genotoxic agent bleomycin sulfate. Extracellular Clusterin promotes epithelial cell apoptosis whereas intracellular Clusterin regulates cellular senescence via the modulation of DNA repair pathways. Based upon the findings in this study, the differential localization of Clusterin is likely to both predispose epithelial cells and their progenitors to enhanced sensitivity to pro-apoptotic signals and/or senescence due to altered expression of DNA repair pathways. The IPF lung contains heterogeneous regions of healthy tissue, active disease and more established disease. As a dysregulated and deficient epithelial wound healing response is observed in IPF, replenishing intracellular Clusterin and limiting the activity of extracellular Clusterin might provide a therapeutic benefit by reducing both apoptotic and senescent pressures and consequently promoting epithelial regeneration. Future studies are warranted to explore the therapeutic potential of variants of Clusterin in IPF.

## Methods

### Patients

Patients with a diagnosis of IPF according to the ERS/ATS consensus statement^38^ were recruited for involvement in these studies. Age-matched normal controls were recruited from the local community.

### Somascan^TM^ analysis

Serum samples from 60 IPF patients (average age 64.4 years) and 30 gender-matched healthy controls (average age 70 years), and 15 COPD patients and 25 age and gender-matched healthy controls were analyzed using the SOMAscan^TM^ platform (SomaLogic Inc., Boulder, CO). Proteomic measurements were performed as previously described^39^.

### Bleomycin model of lung injury and fibrosis

The Clusterin null mice (*CLU−/−*), described previously^40^, were licensed from Cincinnati Children’s Hospital. Female C57BL/6 mice or *CLU−/−* mice received an intratracheal bleomycin challenge (Blenoxane, Sigma, St. Louis, MO) as previously described^41^. Bronchoalveolar lavage (BAL) was taken by flushing the lungs with sterile PBS, serum was prepared from a post-mortem blood draw and whole lung lobes were dissected for histological, immunocytochemical analyses, biochemical and gene analyses.

### Gene expression and ELISA

For single cell samples or for lung tissue analysis, lung biopsy tissue from IPF/UIP patients, NSIP patients, COPD patients or the normal margins of lung tumor resections; or lungs from mice were processed for total RNA using TRIzol reagent (Invitrogen) according to the manufacturer’s instructions. Gene expression levels were quantitated using real time RT-PCR (Applied Biosystems) according to the manufacturer’s protocols. Transcript levels of genes of interest measured by quantitative RT-PCR were normalized to housekeeping gene mRNA. For protein analysis, collagen levels were determined in lung homogenates using an established hydroxyproline assay as previously described^41^. Cytokine levels were measured by MSD analysis (Mesoscale), or specific ELISA to mouse TGFβ1 (R&D Systems), or p21 according to manufacturer’s protocols.

### Isolation of fresh IPF explant cells

IPF lung explants were placed into sterile PBS, washed and transferred into fresh PBS. Tissue was minced and spun at 600-x g for 5 minutes. Supernatants were transferred and mixed with the PBS utilized to wash the explanted lungs. The top layer of the pellet enriched in small cells and RBCs were strained through a 70-µm strainer, the strainer washed several times with DPBS. This procedure was repeated until the RBCs and small cells were removed from the main pellet. The flow through was mixed with the PBS utilized to wash the explanted lungs. The mixed wash/PBS sample was spun down at 400-x g for 5 minutes and the RBCs were lysed using RBC lysis buffer (Biolegend). Cells were then counted and viably frozen down.

### Flow cytometry

Lungs were perfused with sterile PBS via the right ventricle and for analysis of total lung cells, single-cell suspension preparations were generated using a method previously described in detail^42^. Briefly, dissected lungs were finely minced in 15 ml of digestion buffer (RPMI, 5% FCS, 1 mg/ml Liberase (Boehringer Mannheim), 30 mg/ml DNase, Sigma), and enzymatically digested for 30 min at 37 °C and passed through a 70 µm cell strainer to obtain cell suspensions. To exclude dead cells, samples were treated with a Live/Dead Cell stain kit (L34957, Life Technologies) according to manufacturer’s instructions. To block non-specific binding, the single-cell suspensions were incubated with anti-CD16/32 antibodies for 20 min prior to staining. Samples were then analyzed on a LSRFortessa flow cytometer (BD), and analysis and gating performed on FlowJo software (version 10, Tree Star). All antibodies used in flow cytometry were purchased from eBioscience (Hatfield, UK) unless otherwise stated and included anti-CD16/32 (clone 93), anti-CD45 APCCy7 (30-F11) and anti-EpCAM1 (G8.8). For Ki67 proliferation staining a BD Fix and Perm kit was used (554715) as per manufacturer’s instructions and anti-Ki67 conjugated to APC was used (Clone 16A8, Biolegend). For normal and IPF lung explant cell analysis, cells were fixed with 5% formalin on ice for 30 minutes, washed twice with Perm/wash buffer (Biolegend) and blocked with anti-human FC for 15 minutes on ice. After blocking, cells were stained with BV421 conjugated anti-EpCAM (Biolegend), unconjugated anti-MSH2 (Lifespan Biosciences) and/or unconjugated anti-BRCA1 (Lifespan Biosciences) antibodies for 30 minutes at 4 °C. Samples were then washed and stained with fluorescent conjugated secondary antibodies for another 30 minutes at 4 °C, washed and fixed with 5% formalin. All samples were analyzed using a MACSQuant 10 flow cytometer (Miltenyi Biotec) and Flowjo Version 10 (Flowjo, LLC).

### Histology

Formalin-fixed and paraffin-embedded lung sections were stained with hematoxylin and eosin to assess gross morphology or Masson’s trichrome stains to visualize collagen deposition or elastin van Gieson to visualize elastin.

For Clusterin, murine 8-Oxo-dG, p-H2A.X and cleaved Caspase-3 staining, paraffin embedded 3 µm sections were stained using automated IHC robots (For Clusterin: Autostainer; Dako, Glostrup, Denmark; for 8-Oxo-dG and p-H2A.X: Ventana Discovery Ultra, Roche). Briefly, slides were deparaffinized and rehydrated. Antigen retrieval was performed using DAKO’s antigen retrieval buffer (for Clusterin staining) or CC1 antigen retrieval solution for Ventana (Roche, for 8-Oxo-dG and p-H2A.X staining) or Low pH Antigen Unmasking Solution (Vector, H-3300) and basal peroxidase was blocked with 3% Hydrogen Peroxide solution in Methanol. Slides were then blocked with 2.5% horse serum or 2.5% BSA. After blocking, tissues were stained with goat anti-mouse Clusterin (R&D Systems), mouse anti human Clusterin (BD Pharmingen) diluted in PBST, FITC conjugated anti-8-hydroxyguanosine (Abcam), p-H2A.X (Cell Signaling) or rabbit anti-cleaved Caspase-3 (Cell Signaling) antibodies with background reducing components (DAKO) for 1 hour at room temperature. Tissue was then washed and stained with anti-mouse IgG ImmPRESS-HRP reagent (human Clusterin, Vector Labs), anti-goat Ig ImmPRESS-HRP (mouse Clusterin, Vector Labs), rabbit anti FITC (8-Oxo-dG) secondary or DISCOVERY OmniMap anti-rabbit HRP conjugated secondary antibodies (8-Oxo-dG and p-H2A.X). Slides were then developed using DAB + substrate/chromogen (Dako), counter stained with Gill I hematoxylin, dehydrated and mounted. All images were acquired and analyzed using an Aperio Scanscope slide scanner (Genie).

For human normal and IPF lung 8-Oxo-dG, MSH2, MSH6, SSEA4 and BRCA1 staining, slides containing 4 µm paraffin embedded sections were deparaffinized and hydrated by incubating them in two changes of xylene for five min each, followed by 2 changes of 100% ethanol for 3 min each, 70% ethanol for 2 min, 50% ethanol for 2 min, and distilled water for 5 min. Antigen retrieval was performed by incubating the slides in 10 mM Citric acid solution (pH 6.0) in an 80 °C oven overnight. The slides were subsequently washed in PBS and permeabilized in 10% methanol containing 0.4% H_2_O_2_ for 30 min. For 8-Oxo-dG staining, samples were permeabilized in the absence of H_2_O_2_ to prevent nonspecific oxidation. After permeabilization, slides were stained with mouse anti-8-Oxo-dG (Abcam), rabbit anti-MSH2 (Lifespan bio) or rabbit anti-MSH6 (Abcam). All slides were subsequently stained using an anti-rabbit or mouse HRP-DAB cell and tissue staining kit according to the manufacturer’s instructions (R&D systems). For immunofluorescence staining, permeabilized sections were washed, stained with rabbit anti-human BRCA1 (Lifespan Biosciences, Inc.) and mouse anti-SSEA4 (Abcam) antibodies followed by secondary staining with Alexa Flour 488 chicken anti rabbit and Alexa Flour 594 chicken anti mouse antibodies (Life technologies). Slides were then washed and mounted with Prolong Gold anti-fade mounting reagent with DAPI (Life technologies) and analyzed by fluorescence microscopy using a Zeiss fluorescence microscope.

### *In vitro* assays

Fibroblasts were grown and assayed in DMEM media, NHBE were grown and assays performed using bronchial epithelial growth media (BEGM, Cambrex, Lonza). Clusterin gene knockdown was achieved by transducing normal human bronchial epithelial cells (NHBE) or normal human lung fibroblasts (NHLF) from Lonza, with shRNA expressing lentiviruses. The two shRNA expressing lentiviral vectors with promoter-driven puromycin selection marker were purchased from Sigma (TRCN0000300844 and TRCN0000078610). As a control, a non-target shRNA virus from Sigma was also used (SHC016). Virus was generated and concentrated 50 fold by ultracentrifugation. The virus was added to the cells at an MOI = 10 along with 2μg/ml polybrene. Cells were selected for resistance to puromycin prior to *in vitro* use. The ROCK inhibitor Y-27632 (Sigma) was included at a concentration of 10 μM during the transduction and selection process of the NHBEs as previously described^43^. The efficiency of knockdown was determined by mRNA with quantitative real-time PCR (Applied Biosystems; Hs00205254_m1) and by protein with ELISA (RayBiotech).

### RNA Seq Assessments

For SSEA4^+^ and SSEA4^−^ cells, cells were sorted from 2–3 normal and 7 IPF stromal cultures and RNA was extracted using miRNeasy micro kit (Qiagen). A minimum of 700 ng of total RNA was subjected to RNAseq analysis. For NHBE cells, a minimum of 500 ng of total RNA was assessed by RNAseq analysis. Samples were sequenced to at least 10 million reads. Average FPMK values and the ratios from sorted cells were calculated and the data was uploaded onto Ingenuity’s IPA set to consider changes in gene expression of 1.5 fold or greater. Ingenuity canonical pathways and upstream analysis of SSEA4^+^ and SSEA4^−^ was exported and depicted in the supplemental tables. To generate DNA repair pathway heat maps, FPKM values for the various pathway components were mined, samples clustered using Euclidean distance and complete linkage and heat maps were generated using Morpheus (Broad Institute). Raw sequencing datasets have been deposited and is available on to NCBI’s Geo records under the following accession number: GSE103488.

### Fibroblast senescence studies

Induction of cellular senescence using hydrogen peroxide was performed as previously described^44^. In brief, NHLFs were exposed to 600 μM of H_2_O_2_ for 2 hours in complete media. Media was replaced with fresh media and cells were allowed to rest for 4 days. The cells were then exposed to another dose of H_2_O_2_ and allowed to rest for an additional 4 days. The cells were then plated at 10,000 per well in a 96 well plate overnight, after which supernatants were taken, and cells were lysed. Gene expression fold induction was determined after calibration of the expression of genes of interest with GAPDH and normalized to corresponding unstimulated or scramble shRNA control cells where stated. Analysis of supernatants for cytokines was performed by ELISA according to the manufacturer’s instructions (R&D Systems). Cell lysates were quantitatively analyzed for SA-βgal activity (Enzo Life Sciences).

### Statistics

For *in vivo* studies, data were expressed as means ± SEM, and assessed for significance by Student’s *t* test or ANOVA as appropriate. Patient demographics were compared using Student’s t-test or Mann Whitney analysis. Categorical variables were compared using Fisher’s exact test. P values were determined for multiple comparisons using the Bonferroni correction. Values of P ≤ 0.05 (*), P ≤ 0.01 (**),P ≤ 0.005 (***),P ≤ 0.001 (****) were considered significant.

### Study approval

Institutional Review Boards both at Cedars-Sinai Medical Center and the University of Michigan approved all experiments with primary human tissue. Informed consent was obtained from all patients prior to inclusion in the studies described herein. All mouse experiments were conducted according to UK Home Office regulations and approved protocols. All studies were performed in accordance with the relevant guidelines and regulations.

## Electronic supplementary material

Supplemental methods, figures and tables

Table S1

Table S2

Table S3
