# Supplementary material for: Divergent roles for Clusterin in Lung Injury and Repair
Source: Sci Rep. 2017 Nov 13;7:15444. doi: 10.1038/s41598-017-15670-5 (PMC5684342; doi:10.1038/s41598-017-15670-5)
Supplement: Supplementary file 1 — Supplemental methods, figures and tables [file 41598_2017_15670_MOESM1_ESM.doc]

Divergent roles for Clusterin in Lung Injury and Repair

David M. Habiel^1,*^, Ana Camelo^2^, Milena Espindola^1^, Timothy Burwell^3^, Richard Hanna^3^, Elena Miranda^4^, Alan Carruthers^2^, Matthew Bell^2^, Ana Lucia Coelho^1^, Hao Liu^4^, Fernanda Pilataxi^4^, Lori Clarke^5^, Ethan Grant^6^, Arthur Lewis^2^, Bethany Moore^7^, Darryl A. Knight^8^, Cory M. Hogaboam^1,$^ and Lynne A. Murray^2,*, $^

**Affiliations:**

^1^Department of Medicine, Cedars-Sinai Medical Center, Los Angeles, CA, USA; ^2^Respiratory, Inflammation and Autoimmunity, MedImmune Ltd, Cambridge, United Kingdom; ^3^Respiratory, Inflammation and Autoimmunity, MedImmune LLC, Gaithersburg, MD, USA; ^4^Translational Sciences, MedImmune LLC, Gaithersburg, MD, USA; ^5^Molecular Biology, MedImmune LLC, Gaithersburg, MD, USA; ^6^Translational Medicine, MedImmune LLC, Gaithersburg, MD, USA; ^7^Department of Internal Medicine, Division of Pulmonary and Critical Care Medicine, University of Michigan Health System, Ann Arbor, Michigan, USA; ^8^School of Biomedical Sciences and Pharmacy, University of Newcastle, Callaghan, NSW, Australia.

*Correspondence:

Lynne A. Murray Ph.D

MedImmune Ltd,

Granta Park, Cambridge,

CB21 6GH, UK

Telephone +44-1223-898557

e-mail: [murrayl@medimmune.com](mailto:murrayl@medimmune.com)

and

David Habiel, Ph.D.

Cedars-Sinai Medical Center

127 S San Vicente Blvd.

AHSP 9404

Los Angeles, CA 90048

Ph: 1 (424) 315-2376

e-mail: [david.habiel@cshs.org](mailto:david.habiel@cshs.org)

^$^Hogaboam C.M., and Murray L.M. are co-senior authors

**Competing interests**: The authors have declared that no conflict of interest exists.

**Supplemental Methods:**

**Gene expression array data mining and Ingenuity IPA analysis:**

Publicly available gene expression datasets (GSE35309) were mined from NCBI’s geo datasets database. Groups were defined as follows GSE35309: hyperplastic fibroblastic foci adjacent epithelial cells versus normal. Gene expression values were extracted using NCBI’s Geo2R gene expression analysis tool and the expression data were uploaded to and analyzed using ingenuity IPA^1^ (QIAGEN Inc., https://www.qiagenbioinformatics.com/products/ingenuitypathway-analysis). Ingenuity IPA was set to only consider changes in gene expression of 1.5-fold or greater and p ≤ 0.05. Shown is Ingenuity’s Clusterin interaction network, set to only consider direct interactions, and overlaid with the GSE35309 gene expression datasets.

**Elastic Van Gieson staining:**

Paraffin embedded FFPE sections were placed in water and then oxidized in 5% Potassium Permanganate (VWR) for 5 minutes. Sections were then bleached and cleared in 1% Oxalic acid (VWR), washed in distilled water and rinsed in 70% ethanol. The sections were then incubated in Miller’s Elastic stain (VWR) for 60 minutes, then washed in running tap water and counter stained in Harris Haematoxylin for 5 minutes. After staining, sections were differentiated in 1% acid alcohol (70% ethanol) for 10 seconds, transferred into Van Gieson solution (VWR) for 3 minutes and then dehydrated and mounted. All images were acquired and analyzed using an Aperio Scanscope slide scanner (Genie).

**Confocal Imaging of Clusterin Binding and Internalization**

Normal human bronchial epithelial (NHBE) cells (Lonza) were cultured in BEBM media (Lonza) with BEBM supplements (Lonza). Cells were dissociated using StemPro Accutase (Gibco), and transferred to Lab-TekII 8 chamber coverslips (Nunc) at approximately 200k cells in 400ul of BEBM media per chamber. Cells were allowed to rest for 2 days, and then 10ug/ml of A594-labeled clusterin was added per well. Recombinant carrier-free human clusterin (Biolegend) was labeled with A594 using a protein labeling kit following the manufactures directions (Invitrogen). Cell Mask Green (Invitrogen) membrane label (1:1000) and NucBlue live cell stain (Invitrogen) nuclear dye (1:500) were added directly to imaging chambers prior to imaging. NHBE cells were imaged using a Leica SP5 confocal microscope. Clusterin Binding was analyzed by quantifying the number of clusterin particles bound per cell increased for a minimum of 4 hours. At least 15 cells from multiple images for each time point were analyzed using Imaris image analysis software (Bitplane).

**Supplemental Figure Legends:**


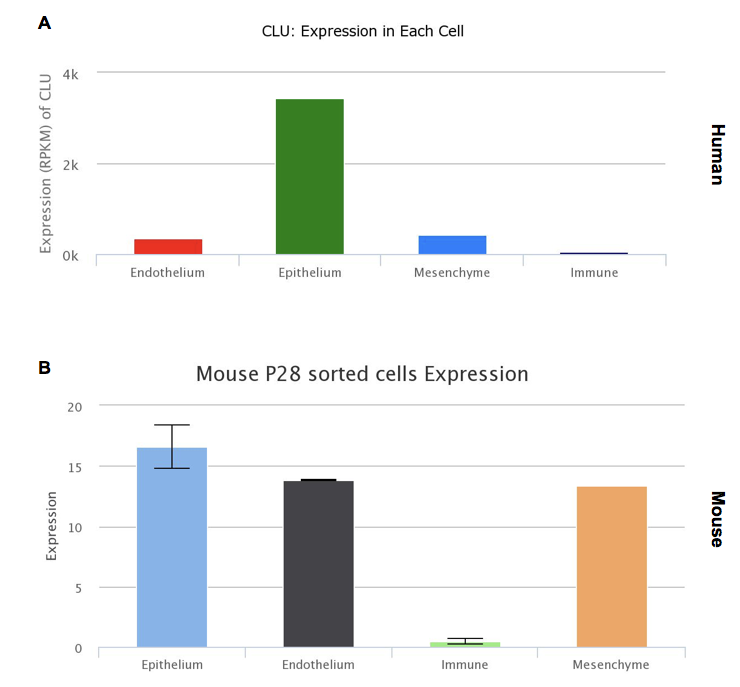


Figure S1: *Clusterin expression by sorted lung associated immune and structural cells.*

**(A-B)** Publicly available RNAseq datasets ^2-4^ of sorted lung-associated immune and structural cells were mined for Clusterin transcript expression. Depicted is the average TPM expression for Clusterin transcript in human (A) and murine (B) lung-associated epithelial, endothelial, mesenchymal and immune cells.

**
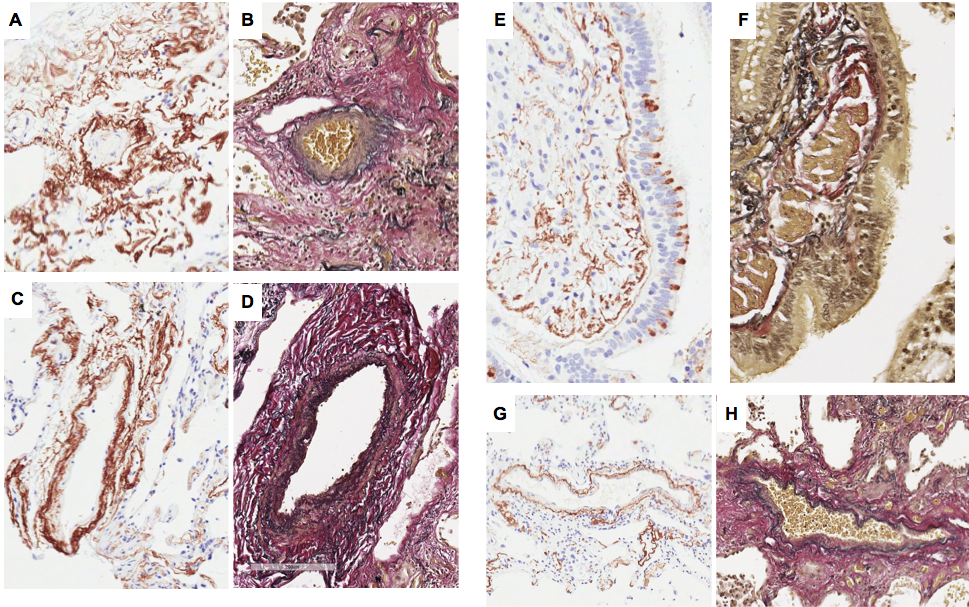
**

Figure S2: *Expression of Clusterin protein in Elastin rich regions of IPF lungs.*

**(A-H)** IPF lung tissue was stained for Clusterin and Elastin (via Elastic Van Gieson staining). Depicted are representative images of Clusterin (A, C, E & G) and Elastin (B, D, F & H) staining on serial sections of fibrotic IPF lung tissue.


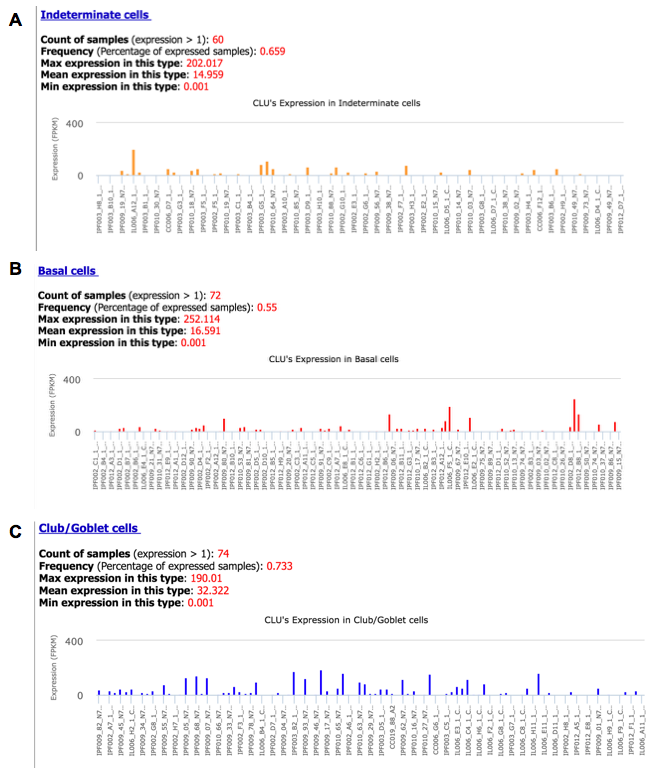


Figure S3: *Clusterin expression in single cell RNAseq datasets of IPF epithelial cells.*

**(A-C)** Publicly available single cell RNAseq datasets^2-4^ of IPF epithelial cells were mined for Clusterin transcript expression. Depicted is the average TPM expression for Clusterin transcript in IPF indeterminate (A), Basal (B) and Club/Goblet (C) cells. Each bar represents the average expression in a single epithelial cell from one IPF patient. n=6 patients analyzed.


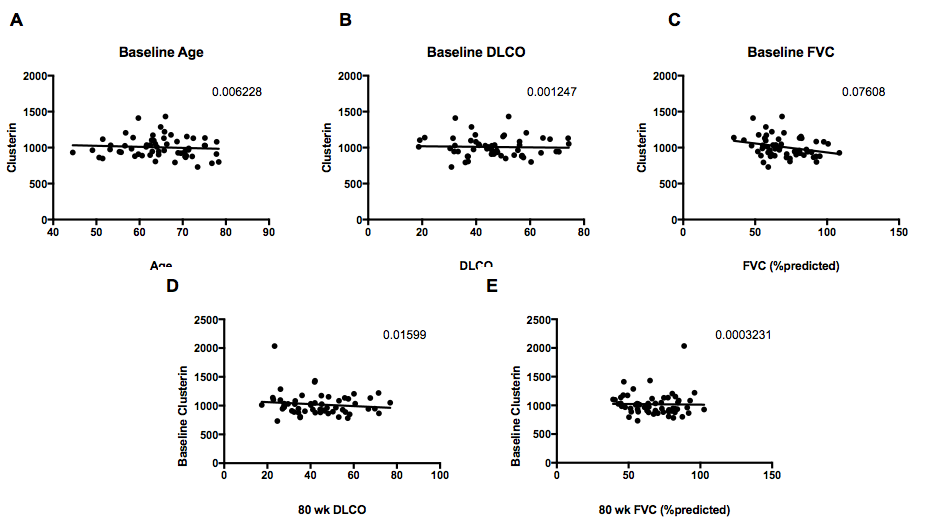


Figure S4: *Correlation between Clusterin protein levels and lung function in IPF patients.*

**(A-C)** Depicted is correlation analysis between baseline Clusterin protein (RU) and Age (A), DLCO (B) or FVC (C) of IPF patients (n=60). **(D-E)** Depicted is correlation analysis between baseline Clusterin protein (RU) and DLCO (D) or FVC (E) as measured at 80 weeks after the enrollment of IPF patients (n=57) into the COMET study. Clusterin protein levels were measured by Somascan analysis, each dot representing a different individual.


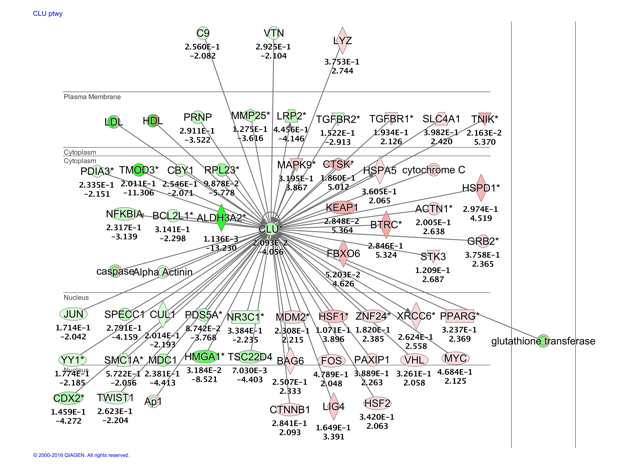


Figure S5: *Clusterin and its cell associated interacting protein are downregulated in fibroblastic foci adjacent hyperplastic epithelial cells.*

Publicly available gene expression datasets (GSE35309) were mined from NCBI’s geo datasets database. Gene expression values were extracted using NCBI’s Geo2R gene expression analysis tool and the expression data were uploaded onto ingenuity IPA^1^ (QIAGEN Inc., https://www.qiagenbioinformatics.com/products/ingenuitypathway-analysis). Shown is Ingenuity’s Clusterin interaction network, set to only consider direct interactions, and overlaid with the GSE35309 gene expression fold change (bottom) and p-value (top). Red – upregulated transcripts by ≥1.5 fold and a p-value ≤0.05; Green - downregulated transcripts by ≥1.5 fold and a p-value ≤0.05.


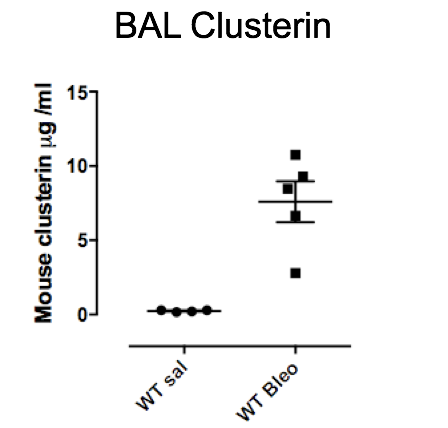


Figure S6: *BAL Clusterin concentration in murine lungs, 14 days after bleomycin treatment.*

Clusterin protein concentration in the BAL of saline and bleomycin treated mice was determined via ELISA. Depicted is the average BAL protein concentration, 14 days after saline or bleomycin treatment of wildtype C57bl/6 mice. n=4-5/group.


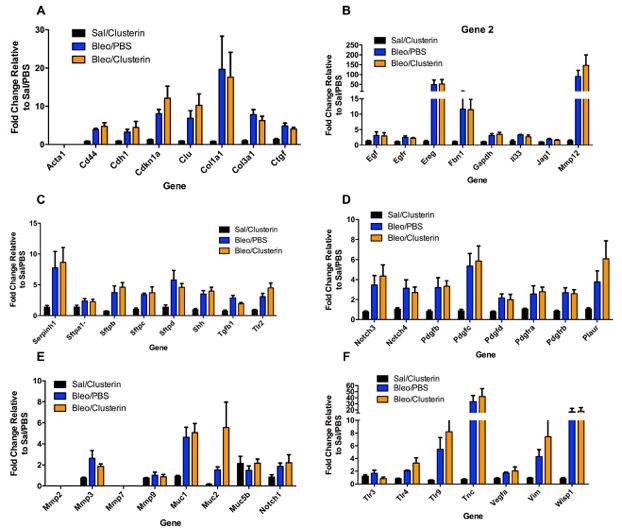


*Figure S7: Transcriptomic analysis of recombinant Clusterin treated bleomycin challenged mice.*

Saline- or bleomycin-challenged C57Bl/6 were treated with 20µg of recombinant Clusterin every three days starting at Day 2. **(A-F)** Various transcript expression were quantified in whole lung samples 15 days after bleomycin, vehicle and/or Clusterin treatment. Shown is the average transcript expression from 3 Saline/PBS, 3 Saline/rClusterin, 7 Bleo/PBS and 7 Bleo/rClusterin treated mice.


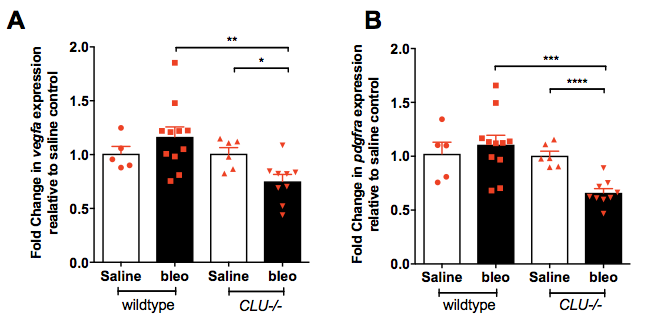


Figure S8: *CLU-/- mice show reduced Vegfa and Pdgfra transcript expression 28 days after bleomycin challenge.*

**(A-B)** Shown is the *Vegfa* (A) and *Pdgfra* (B) transcript expression in whole lungs from wildtype and *CLU-/-* mice, 28 days after bleomycin challenge. Data are Mean±S.E.M., n=8-13 mice/ group. * P≤0.05, **P≤0.01, ***P≤0.005, ****P≤0.001 significance, or as stated. ns=not significant.


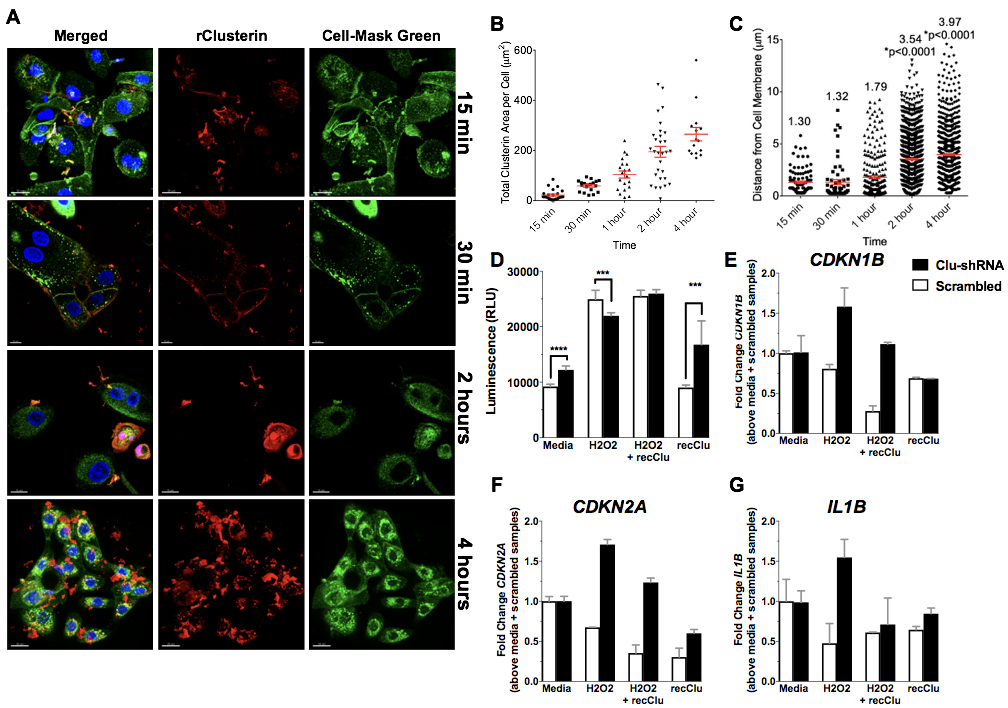


Figure S9: *Clusterin modulation of Caspase 3/7 activity and senescence in primary human bronchial epithelial cells.*

Clusterin expression was knocked down with shRNA in Normal Human Bronchial Epithelial cells (HBECs) and the cells were subsequently exposed to H_2_O_2_ to induce oxidative stress in the presence or absence of rClu protein (10 µg) for 24 h. **(A-C)** Depicted are representative immunofluorescence images showing the uptake of exogenously added Alexa Fluor 594-labeled rClu protein by epithelial cells (A), the number of clusterin particles bound per cell (B) and the distance of bound clusterin from the cell membrane over 4 hours. **(D)** Shown is the average caspase 3/7 activity, as measured using a luminescence Caspase 3/7 activity kit according to the manufacturer’s instructions. Data are expressed as mean± S.E.M. of the luminescence detected. n=2 **(E-G)** Depicted is the average expression of CDKN1B (E) and CDKN2A (F) and IL1B (G) transcripts in HBECs, 24 hours after vehicle (media) or H2O2 treatment. Shown is the average from 2 replicates.


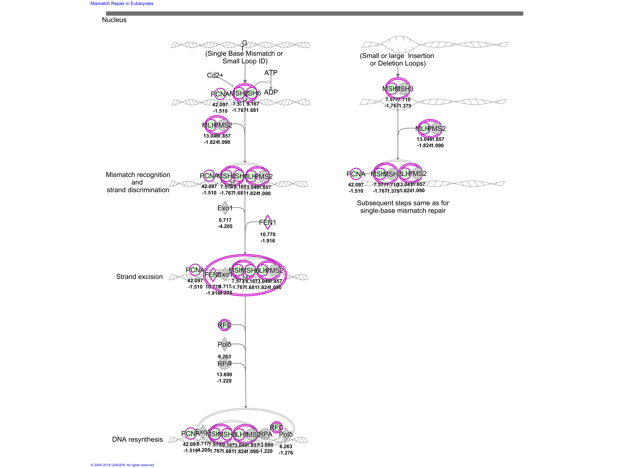


Figure S10: *Loss of various components from the Mismatch Repair in Eukaryotes Ingenuity Canonical pathway in after Clusterin knockdown in lung epithelial cells.*

Clusterin expression was knocked down with shRNA in lung epithelial cells, RNA was extracted and subjected to RNAseq analysis. Fold change expression values were generated relative to RNAseq analysis generated from scrambled shRNA treated lung epithelial cell controls. The data were then uploaded onto Ingenuity IPA^1^ (QIAGEN Inc., https://www.qiagenbioinformatics.com/products/ingenuitypathway-analysis), analysis was set to consider an experimental intensity of ≥2 and a fold change ≥1.5. Depicted is the Mismatch repair in Eukaryotes Ingenuity Canonical Pathway showing upregulated (red) and downregulated transcripts (green) with the expression counts in the knockdown cells (Top), the fold change in expression relative to scrambled shRNA treated epithelial cells (Bottom).


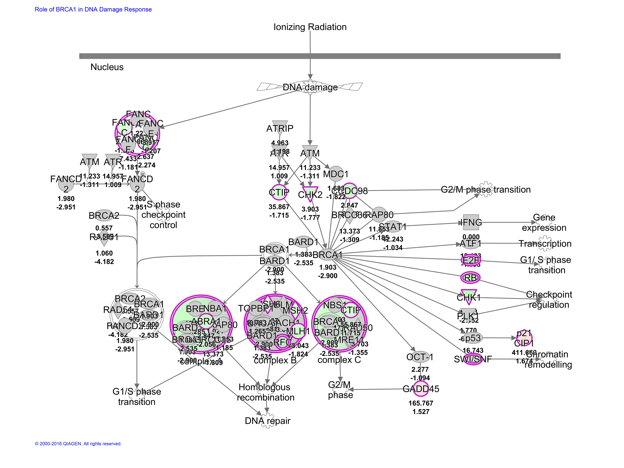


Figure S11: *Loss of various components from Role of BRCA1 in DNA Damage Response Ingenuity Canonical pathway in after Clusterin knockdown in lung epithelial cells.*

Clusterin expression was knocked down with shRNA in lung epithelial cells, RNA was extracted and subjected to RNAseq analysis. Fold change expression values were generated relative to RNAseq analysis generated from scrambled shRNA treated lung epithelial cell controls. The data were then uploaded onto Ingenuity IPA^1^ (QIAGEN Inc., https://www.qiagenbioinformatics.com/products/ingenuitypathway-analysis); analysis was set to consider an experimental intensity of ≥2 and a fold change ≥1.5. Depicted is the Role of BRCA1 in DNA Damage Response Ingenuity Canonical Pathway showing upregulated (red) and downregulated transcripts (green) with the expression counts in the knockdown cells (Top), the fold change in expression relative to scrambled shRNA treated epithelial cells (Bottom).


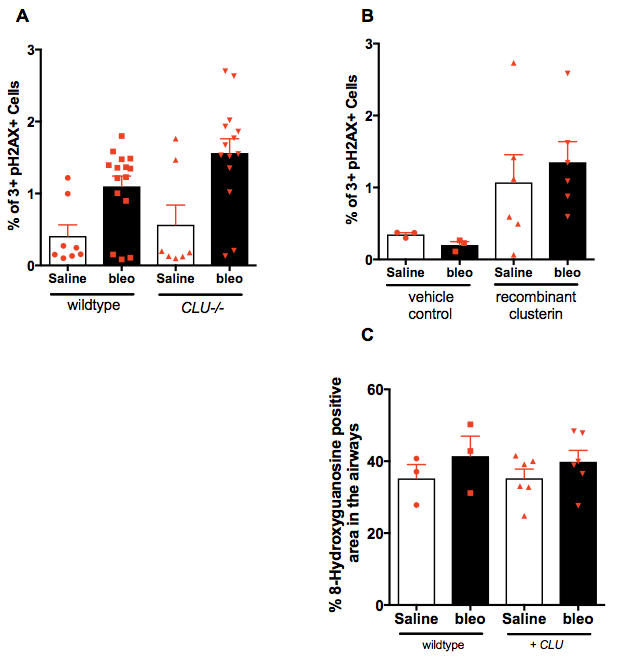


Figure S12: *DNA damage analysis in wildtype, saline or Clusterin treated, and CLU-/- mice, 14 days after saline or bleomycin treatment.*

Fourteen-days after bleomycin or saline administration in wildtype and *CLU-/-* mice, lungs were histologically stained for various markers for DNA damage. **(A-B)** Depicted is the percent of cell clusters, as defined by three or more adjacent positive cells, staining for p-H2A.X, as determined using Aperio Scanscope software in saline and bleomycin-challenged wildtype and *CLU-/-* mice (A) and Clusterin treated saline or bleomycin challenged wildtype mice (B). **(C)** 8-Oxo-dG staining intensity in airway epithelial cells as determined using Aperio Scanscope software in Clusterin treated saline and bleomycin challenged wildtype mice.

Figure S13: *Transcript expression of sftpc in saline or bleomycin treated wildtype and CLU-/- mice.*

Quantitative PCR analysis was performed on RNA extracted from the lungs of saline and bleomycin treated wildtype and *CLU-/-* mice. Depicted is the average fold change of *sftpc* transcript expression in bleomycin treated mice relative to the appropriate saline control. n=6-8/group.

**Supplemental Table Legends:**

Table S1: *shRNA mediated Clusterin knockdown leads to a loss of various DNA repair associated transcripts.*

Clusterin expression was knocked down with shRNA in lung epithelial cells, RNA was extracted and subjected to RNAseq analysis. Fold change expression values were generated relative to RNAseq analysis generated from scrambled shRNA treated lung epithelial cell controls. The data were then uploaded onto Ingenuity IPA^1^ (QIAGEN Inc., https://www.qiagenbioinformatics.com/products/ingenuitypathway-analysis), analysis was set to consider an experimental intensity of ≥2 and a fold change ≥1.5. Depicted is a table showing the top 20 enriched Ingenuity Canonical Pathways whose activity based on ingenuity’s Z score or component expression was increased (red) or decreased (green).

Table S2: *shRNA mediated Clusterin knockdown leads to an enrichment of senescence associated upstream regulators.*

Clusterin expression was knocked down with shRNA in lung epithelial cells, RNA was extracted and subjected to RNAseq analysis. Fold change expression values were generated relative to RNAseq analysis generated from scrambled shRNA treated lung epithelial cell controls. The data were then uploaded onto Ingenuity IPA^1^ (QIAGEN Inc., https://www.qiagenbioinformatics.com/products/ingenuitypathway-analysis), analysis was set to consider an experimental intensity of ≥2 and a fold change ≥1.5. Depicted is a table showing the top 20 Upstream Regulators, predicted to be activated in cells treated with shRNA targeting Clusterin relative to their scrambled shRNA treated controls, sorted according to the activation z-score. Senescence associated upstream regulators are depicted in red.

Table S3: *Ingenuity Upstream analysis of IPF vs. Normal SSEA4^+^ shows an enrichment of senescent associated upstream regulators.*

SSEA4^+^ cells were sorted from Normal and IPF stromal cultures, RNA was extracted and subjected to RNAseq analysis. Fold changes were calculated from the resulting FPKM values and the resulting data were then uploaded onto Ingenuity IPA^1^ (QIAGEN Inc., https://www.qiagenbioinformatics.com/products/ingenuitypathway-analysis), analysis were set to consider an experimental intensity of ≥2 and a fold change ≥1.5. Shown above are upstream regulators predicted to be activated using Ingenuity’s upstream analysis in IPF vs. Normal SSEA4^+^ cells. Senescence associated upstream regulators are depicted in red.

**Supplemental References:**

1 Kramer, A., Green, J., Pollard, J., Jr. & Tugendreich, S. Causal analysis approaches in Ingenuity Pathway Analysis. *Bioinformatics* **30**, 523-530, doi:10.1093/bioinformatics/btt703 (2014).

2 Guo, M., Wang, H., Potter, S. S., Whitsett, J. A. & Xu, Y. SINCERA: A Pipeline for Single-Cell RNA-Seq Profiling Analysis. *PLoS Comput Biol* **11**, e1004575, doi:10.1371/journal.pcbi.1004575 (2015).

3 Du, Y., Guo, M., Whitsett, J. A. & Xu, Y. 'LungGENS': a web-based tool for mapping single-cell gene expression in the developing lung. *Thorax* **70**, 1092-1094, doi:10.1136/thoraxjnl-2015-207035 (2015).

4 Du, Y. *et al.* Lung Gene Expression Analysis (LGEA): an integrative web portal for comprehensive gene expression data analysis in lung development. *Thorax* **72**, 481-484, doi:10.1136/thoraxjnl-2016-209598 (2017).
